# Heparan sulfate proteoglycans in beta cells provide a critical link between endoplasmic reticulum stress, oxidative stress and type 2 diabetes

**DOI:** 10.1371/journal.pone.0252607

**Published:** 2021-06-04

**Authors:** Sarita Dhounchak, Sarah K. Popp, Debra J. Brown, D. Ross Laybutt, Trevor J. Biden, Stefan R. Bornstein, Christopher R. Parish, Charmaine J. Simeonovic

**Affiliations:** 1 Department of Immunology and Infectious Disease, The John Curtin School of Medical Research, The Australian National University, Canberra, Australian Capital Territory, Australia; 2 Garvan Institute of Medical Research, St Vincent’s Clinical School, The University of NSW (UNSW), Sydney, New South Wales, Australia; 3 Department of Internal Medicine III, Carl Gustav Carus Medical School, Technical University of Dresden, Dresden, Germany; 4 ACRF Department of Cancer Biology and Therapeutics, The John Curtin School of Medical Research, The Australian National University, Canberra, Australian Capital Territory, Australia; Universite Paris Diderot-Paris7 - Batiment des Grands Moulins, FRANCE

## Abstract

Heparan sulfate proteoglycans (HSPGs) consist of a core protein with side chains of the glycosaminoglycan heparan sulfate (HS). We have previously identified (i) the HSPGs syndecan-1 (SDC1), and collagen type XVIII (COL18) inside mouse and human islet beta cells, and (ii) a critical role for HS in beta cell survival and protection from reactive oxygen species (ROS). The objective of this study was to investigate whether endoplasmic reticulum (ER) stress contributes to oxidative stress and type 2 diabetes (T2D) by depleting beta cell HSPGs/HS. A rapid loss of intra-islet/beta cell HSPGs, HS and heparanase (HPSE, an HS-degrading enzyme) accompanied upregulation of islet ER stress gene expression in both young T2D-prone db/db and Akita Ins2^WT/C96Y^ mice. In MIN6 beta cells, HSPGs, HS and HPSE were reduced following treatment with pharmacological inducers of ER stress (thapsigargin or tunicamycin). Treatment of young db/db mice with Tauroursodeoxycholic acid (TUDCA), a chemical protein folding chaperone that relieves ER stress, improved glycemic control and increased intra-islet HSPG/HS. *In vitro*, HS replacement with heparin (a highly sulfated HS analogue) significantly increased the survival of wild-type and db/db beta cells and restored their resistance to hydrogen peroxide-induced death. We conclude that ER stress inhibits the synthesis/maturation of HSPG core proteins which are essential for HS assembly, thereby exacerbating oxidative stress and promoting beta cell failure. Diminished intracellular HSPGs/HS represent a previously unrecognized critical link bridging ER stress, oxidative stress and beta cell failure in T2D.

## Introduction

Type 2 diabetes (T2D) is a metabolic syndrome disease in which obesity, insulin resistance, hyperglycemia, dyslipidemia and chronic low-grade inflammation of pancreatic islets contribute directly or indirectly to beta cell failure [1, 2]. Under normal conditions, pancreatic beta cells are highly metabolically active, due to their specialized role in synthesizing proinsulin/insulin. The ER is a subcellular compartment that functions to chemically modify and fold newly synthesized proteins, including proinsulin, into their mature state. Metabolic dysfunction in T2D and insulin (*INS*) gene mutations disrupt ER homeostasis, resulting in the accumulation of misfolded proteins in the ER lumen and ER stress [3–5]. ER stress is exacerbated by the reduced availability of protein-folding chaperones, inhibition of protein glycosylation, depletion of calcium ions from the ER and local oxidative stress [6, 7]. The unfolded protein response (UPR) is an adaptive response for relieving ER stress by decreasing “global” protein synthesis (at the level of translation) and increasing the transcription of protective genes e.g., ER chaperones [6, 7]. The capacity for the UPR to prolong beta function is determined by a delicate balance between these two processes.

Oxidative stress is also a key disorder outside the ER in T2D beta cells and is a consequence of increased metabolic demand [5, 8–10]. Under normoglycemic conditions, beta cells produce ROS at multiple sites by (i) oxidative phosphorylation in mitochondria, (ii) disulfide bond formation during proinsulin protein folding in the ER [11] and (iii) NADPH oxidase, localized at the cell membrane and in insulin granules [8, 12]. Neutralization of intracellular ROS is an essential process for preventing oxidative damage and preserving beta cell integrity. However, beta cells have low basal levels of antioxidant enzymes, relative to other tissues [13, 14], suggesting that a compensatory antioxidant mechanism exists. Recently we discovered that normal mouse and human beta cells have an unusually high intracellular level of highly sulfated heparan sulfate (HS), which is critical for their survival [15, 16]. Mechanistically, beta cell HS provides a constitutive mechanism for quenching intracellular ROS generated during normal metabolism [15, 16]. In T2D, oxidative stress and ER stress contribute to beta cell failure [4–6, 17–19], with elevated ROS representing a mediator or by-product, respectively, in different sub-cellular compartments [5]. This dysregulation of ROS suggests that the antioxidant capacity of HS is impaired in beta cells in T2D.

HS is a sulfated linear polysaccharide containing repeating disaccharides of *N*-acetylated glucosamine and uronic acid [20, 21]. HS chains are synthesized directly onto a core protein forming a HSPG, e.g., collagen type XVIII (COL18), syndecans (SDC1-4), glypicans (1–6), agrin, CD44 and perlecan [20–24]. The core proteins are essential to HS synthesis because they provide the necessary serine residues required to initiate HS assembly [23]. While HSPG core proteins undergo folding and maturation in the ER, HS chains are assembled onto the core proteins in the Golgi apparatus and subsequently undergo chemical modification [21–23]. Conventionally HSPGs are expressed extracellularly where their HS chains function in cell adhesion, as reservoirs for bioactive proteins and as structural components in basement membranes [21–26]. In contrast, HSPG core proteins (COL18, SDC1, CD44)/HS are highly expressed intracellularly in normal mouse and human beta cells [15, 16, 27]. HS in beta cells functions as a non-enzymatic antioxidant [15, 16, 28], influences postnatal islet maturation and regulates insulin secretion [29, 30]. In addition, studies in beta cell lines have also demonstrated that desulfation of HS impairs protection from hydrogen peroxide-induced death [31] and that the HSPG core protein syndecan (SDC4) plays an important role in insulin secretion [32].

Beta cell HS deteriorates acutely during the isolation of normal islets *in vitro* and chronically during the development of type 1 diabetes (T1D), due to excessive oxidative damage and HS degradation by leukocyte-derived HPSE, respectively [15, 16]. *In vitro*, replacement of lost HS using HS mimetics preserves the survival of isolated beta cells and provides protection against hydrogen peroxide (ROS)-induced cell death [15, 16]. Supporting a mechanistic role for HPSE in T1D, *in vivo* treatment of NOD mice with PI-88 (a heparanase inhibitor/HS replacer), reduced the incidence of diabetes, improved islet pathology and preserved beta cell HS [15]. We also found that beta cell HSPG core proteins were decreased in human pancreases from donors with T1D [16], potentially due to protease(s) produced by invading leukocytes and/or ER stress [33–35]. We reason that the substantial loss of beta cell HS following islet/beta cell isolation *in vitro* and in T1D increases the sensitivity of beta cells to oxidative (ROS-mediated) damage [15, 16, 28]. Against this background we have explored whether beta cell HSPGs and HS are altered in T2D and impact cell viability.

In this study we investigate the relationship between spontaneous ER stress and beta cell HSPGs/HS in the db/db mouse model of obesity-induced T2D and in Akita (Ins2^WT/C96Y^) mice. In addition, we experimentally induce ER stress in MIN6 beta cells *in vitro* and monitor intracellular HSPGs/HS. Intervention studies further interrogate the relationship between (i) ER stress and beta cell HSPGs/HS in db/db mice treated with TUDCA to relieve ER stress and (ii) intracellular HS, oxidative stress and the viability of db/db beta cells *in vitro* using HS replacement and acute exposure to hydrogen peroxide. Our findings reveal a previously unrecognized inter-relationship between ER stress, HSPGs, HS and oxidative stress in beta cells.

## Materials and methods

### Animals

C57BL/KsJ db/db, heterozygous (db/+) and wild-type (wt; +/+) mice, heterozygous Akita (Ins2^WT/C96Y^) and wild-type Ins2^WT/WT^ mice were obtained from the Australian Phenomics Facility, The Australian National University, Canberra, ACT, Australia. Db/db mice carry a homozygous point mutation in the leptin receptor gene and develop obesity and hyperglycemia [36]; heterozygous Akita mice have a point mutation in the insulin 2 (*Ins2*) gene, resulting in misfolding of proinsulin, ER stress in beta cells and rapid onset of diabetes [37]. The mice were housed under specific pathogen free conditions in individually ventilated cages (up to 5 mice/cage). All mice were maintained with irradiated standard rodent chow and water ad libitum with environmental conditions set at ~ 19°C and an automated 12 hr light/dark cycle. Groups of male db/db mice at 4 weeks of age were randomly selected and treated daily (a.m.) with TUDCA (Tauroursodeoxycholic acid, 150 mg/kg; Calbiochem, EMD Millipore, Darmstadt, Germany) i.p. for 4 weeks [38, 39]; control mice were treated with 8 μl/g saline (diluent) i.p. Non-fasting blood glucose levels and body weight were monitored up to 3x/week (a.m.). After termination of treatment, HbA1c levels were measured using a HemoCue HbA1c 501 analyzer (Angelholm, Sweden). Mice were euthanized by cervical dislocation at the completion of treatment and/or for pancreas harvest. All procedures and experiments were approved by The Australian National University Animal Experimentation Ethics Committee.

### Islet isolation

Pancreatic islets were isolated by intra-ductal infusion of collagenase P (2.5 mg/mL; Roche Diagnostics, Mannheim, Germany) followed by hand-picking [15, 27, 40, 41]. For Akita donors, digested pancreases were resuspended in a dithizone solution (~ 3ml at 20 mg/0.6ml; Sigma-Aldrich, St Louis, MO, USA) to identify islets for hand-picking [42, 43].

### Preparation and culture of isolated mouse islet cells and MIN6 cells

Isolated mouse islets were dispersed into single cells by Accutase (Milllipore, Temecula, CA, USA; 250 μl/500 islets; [16]) (https://dx.doi.org/10.17504/protocols.io.bmgjk3un) and transferred to a 96 well plate (#650180, CELLSTAR, Greiner Bio-one, Frickenhausen, Germany) for staining and analysis by flow cytometry or for culture. Islet cells (> 90% beta cells [15]) were cultured in RPMI 1640 media supplemented with 10% heat-inactivated fetal calf serum (HIFCS; Sigma-Aldrich, St. Louis, MO, USA), L-glutamine (Life Technologies, Carlsbad, CA, USA) and antibiotics (80.7 mM Penicillin G (MP Biomedicals, Santa Ana, CA, USA), 68.6 mM Streptomycin sulfate (Sigma-Aldrich), 5 mM Neomycin sulfate (Sigma-Aldrich)) in a 96 well plate (4 x 10^4^ cells/well) at 37°C in humidified 5% CO_2_/ 95% air. MIN6 beta cells (authenticated/screened and provided by R. Laybutt/T. Biden, The Garvan Institute of Medical Research, Sydney) [44] were maintained in culture in DMEM with high glucose (25 mM; GIBCO-Invitrogen, Carlsbad, CA, USA) supplemented with 10% HIFCS, 15 mM HEPES (GIBCO-Invitrogen) and antibiotics at 37°C in humidified 5% CO_2_/ 95% air. MIN6 cells were were treated in culture with thapsigargin (50 nM; Sigma-Aldrich) or tunicamycin (2 μM; Sigma-Aldrich) in 6 mM glucose DMEM medium for 20–24 hrs for ER stress assessment by qRT-PCR or for 3 days for analysis of HSPG core proteins, HS and HPSE by flow cytometry.

### Real-time RT-PCR

Two-step qRT-PCR was used to measure transcripts of ER stress markers (*Bip*, *p58*, *Chop* and *Atf3*) in isolated islets and MIN6 cells. Total RNA was extracted from isolated islets (100–743 islets) using the RNeasy kit (Qiagen, Hilden, Germany) and cDNA was synthesized using Superscript RNase reverse transcriptase III (Invitrogen, Carlsbad, CA, USA). Real-time PCR was performed using TaqMan gene expression assays with FAM-labelled TaqMan^®^ MGB probe (ThermoFisher, Waltham, MA, USA; S1 Table) and a AB17900HT real-time PCR machine with 7900HT Sequence Detection system (v2.2.2) software (Applied Biosystems, Foster City, CA, USA). Gene expression was quantified using the comparative C_T_ method [15, 27, 45] with values normalized to the house-keeping gene *Gapdh* (for islets) or *Ube2d1* (for MIN6 cells) and expressed as a fold change compared to control samples (control MIN6 cells, Ins2^WT/WT^ islets, wt or db/+ islets or mouse kidney (irrelevant tissue) samples).

### Culture of primary beta cells with HS mimetics

Isolated mouse islet cells were cultured ± heparin (an HS analogue; Celsus Laboratories, Cincinnati, OH, USA; 50 μg/ml) or PI-88 (a HS mimetic; Muparfostat; Progen Pharmaceuticals, Brisbane, Australia; 50 μg/ml) in 96 well plates (4 x 10^4^ cells/well) for 2 days at 37°C [15, 16] followed by assessment of beta cell viability by flow cytometry analysis.

### Flow cytometry

Primary beta cells (4 x 10^4^ cells/well) and MIN6 cells (2 x 10^5^ cells/well) were stained either for cell surface or intracellular expression of HSPGs, HS and HPSE in separate aliquots of the same cell population [27]. Briefly, primary beta cells and MIN6 beta cells were fixed and permeabilized using the CytoFix/Cytoperm Fixation/Permeabilization kit (BD Biosciences, #554722) for intracellular staining. For either cell surface or intracellular staining, the cells were incubated with Fc block (rat anti-mouse CD16/CD32 mAb) for 10 mins on ice followed by unlabelled primary antibodies (mouse anti-mouse collagen type XVIII (COL18)), rat anti-mouse CD138 (anti-syndecan-1 (SDC1)), rat anti-mouse CD44, 10E4 mouse anti-human HS or HP3/17 mouse anti-human heparanase (HPSE)) for 30 mins on ice (S2 Table). The cells were washed with either 10% FCS/PBS (cell surface staining) or BD Perm/Wash buffer (CytoFix/Cytoperm Fixation/Permeabilization kit; intracellular staining) and incubated with secondary antibodies (goat anti-mouse Ig PE, mouse anti-rat kappa PE or rat anti-mouse Ig FITC) for 30 mins on ice (S2 Table; https://dx.doi.org/10.17504/protocols.io.bmsyk6fw). The stained cells were transferred to FACs mini tubes (Axygen, Salt Lake City, UT, USA) and run on a flow cytometer. The geometric mean fluorescence intensity (GMFI) of staining was determined using FlowJo software (version 10.0.7, TreeStar Inc., Ashland, OR, USA). The viability of primary beta cells was assessed by labelling with 0.04 μM Calcein-AM (Life Technologies, Eugene, OR, USA) followed by 2.5 μg/ml propidium iodide (PI; BD Biosciences, San Jose, CA, USA) for 15 min at 37°C [15, 16]. Beta cell death ± treatment with 30% H_2_O_2_ (Chem-Supply, Gillman, South Australia, Australia) was analyzed by labelling with Sytox Green (31.25 nM; Life Technologies) for 15 mins [15, 16]. Flow Jo software (version 10.0.7) was used to determine % viable cells (Cal^+^PI^-^), dead cells (Cal^-^PI^+^) and damaged cells (Cal^+^PI^+^) or % Sytox Green+ve dead/damaged cells (https://dx.doi.org/10.17504/protocols.io.bmgjk3un).

### Histology and immunohistochemistry

Pancreases were fixed in 10% neutral-buffered formalin, and paraffin sections (4 μm) were stained with haematoxylin and eosin (H&E) or by immunohistochemistry. For immunohistochemistry, antigen retrieval for HS and COL18 was performed using 0.05% pronase (Calbiochem) whereas heat/citrate buffer (pH 6) was used for SDC1 and CD44 [16, 27]. HS was detected immunohistochemically using either 10E4 mouse anti-human HS (recognizes N-sulfated/N-acetylated glucosamine in HS; [31, 46, 47]) or VSV-tagged phage display single chain variable fragment (scFv) EV3C3 anti-HS Ab (stains for N-sulfation, C5 epimerisation and 2-O sulfation in HS chains; [31, 48, 49]) followed by HRP-conjugated polyclonal rabbit anti-mouse Ig (dx.doi.org/10.17504/protocols.io.bmgbk3sn) or rabbit anti-VSV-G pAb/HRP-conjugated polyclonal swine anti-rabbit Ig, respectively (S3 Table). HSPG core proteins were identified using mouse anti-mouse COL18 (https://dx.doi.org/10.17504/protocols.io.bmgbk3sn), rat anti-mouse SDC1 (CD138; (https://dx.doi.org/10.17504/protocols.io.bmghk3t6) and rat anti-mouse CD44 mAbs (https://dx.doi.org/10.17504/protocols.io.bmgfk3tn), with HRP-conjugated rabbit anti-mouse Ig or anti-rat Ig (Dako, Carpinteria, USA) (S3 Table). For CD44 staining, rat anti-mouse CD44 (IM7) mAb which recognizes all CD44 isoforms [50–53] and mouse anti-human CD44v3 mAb which recognizes a CD44 isoform that carries HS side-chains [50–52], demonstrated a similar pattern of intra-islet staining (S1 Fig). CD44 core protein analyzed using rat anti-mouse CD44 (IM7) mAb in mouse islets therefore represents an HSPG. Insulin was detected (without antigen retrieval) using mouse anti-insulin mAb (S3 Table) with biotinylated anti-mouse IgG and Avidin-Biotin-complex (ABC) reagent [16] (https://dx.doi.org/10.17504/protocols.io.kv6cw9e). Isotype control mouse IgMκ (for anti-HS 10E4 mAb), rat IgG_2ak_, rat IgG_2bk_ or mouse IgG_2b_ (for anti-HSPG mAbs), mouse IgG_1κ_ (for anti-insulin mAb) or rabbit anti-VSV pAb alone (for anti-HS EV3C3 scFv) were used to detect background staining. 3-amino-9-ethylcarbazole (AEC; Sigma-Aldrich) was used as the chromogen. Immunostained pancreas sections were deidentified for blinded analysis and the % of islet area stained was assessed using Image J software with colour deconvolution plugin (v 1.44a; NIH) [16].

### Immunofluorescence staining

For colocalization studies of COL18, paraffin sections were treated with 0.05% pronase for antigen retrieval, blocked with M.O.M Ig block in 2% bovine serum albumin (BSA; Sigma)/phosphate buffered saline (PBS) and incubated overnight (4° C) with COL18A1 mAb (1/50). After washing, the sections were then stained with AlexaFluor 488-donkey anti-mouse IgG (Thermo Fisher, Rockford, IL, USA). The sections were washed and then incubated with rabbit anti-human glucagon IgG (Abcam, Cambridge, UK) or guinea-pig anti-insulin Ig (Dako, Santa Clara, CA, USA). Following washing, the sections were stained with AlexaFluor 568-donkey anti-rabbit IgG or AlexaFluor 568-goat anti-guinea-pig IgG (Thermo Fisher) (https://dx.doi.org/10.17504/protocols.io.btqynmxw). Background staining was determined using sections stained only with the secondary antibody. Nuclei were stained with DAPI (0.2 μg/ml; Sigma). After washing, Trueblack (Biotium, Fremont, CA; 1/20 in 70% ethanol) was applied for 30 secs to reduce autofluorescence. The slides were washed and mounted using a 1.5H cover glass (Marienfeld, #0107222, Lauda-Konigshofen, Germany) and antifade mounting medium (ProLong Diamond, Invitrogen). Sections were imaged using an automated Axio Observer inverted fluorescence microscope (Zeiss; Göttingen, Germany). Merged images were prepared using ZEN (version 2.3) software (Zeiss).

### Statistical analysis

Data are presented as mean ± SEM. Statistical analyses included one-way ANOVA with Fischer’s unprotected least significant difference (LSD), unpaired t-test and Mann-Whitney test.

## Results

### Age-dependent decline in intracellular HSPG core proteins and HS in db/db islets

**Fig 1:**
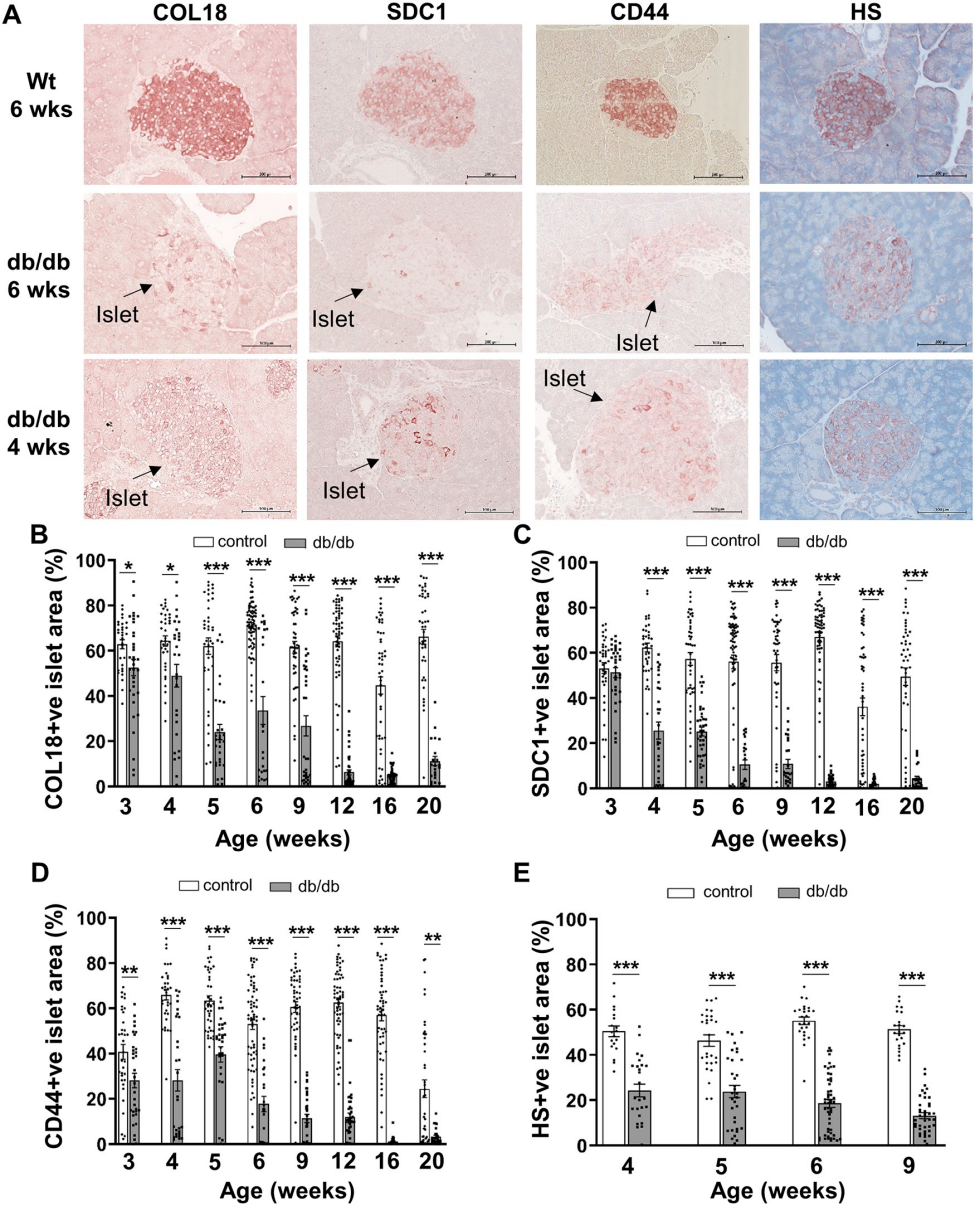
HSPG core proteins and HS rapidly decline in the pancreatic islets of db/db mice. (**A**) Representative images show the distribution of HSPG core proteins and HS in the islets of male db/db mice and wt mice at 4 and/or 6 weeks of age as demonstrated by immunohistochemical staining. (**B-E**) Bar graphs for each time point show morphometric analysis of the % islet stained for the HSPG core proteins (**B**) COL18, (**C**) SDC1, (**D**) CD44 and HS (**E**) in pancreases of lean control male mice (wt, db/+; open bars) and db/db mice (shaded bars). Data show mean ± SEM for 3–6 pancreases/age group with n = 21–72 islets examined/group for HSPG core proteins and n = 19–52 islets/group for HS. *p<0.05, **p<0.01 and ***p<0.0001, Mann-Whitney test (HSPGs) and Unpaired t test (HS). Scale bar = 100 μm.

**Fig 2:**
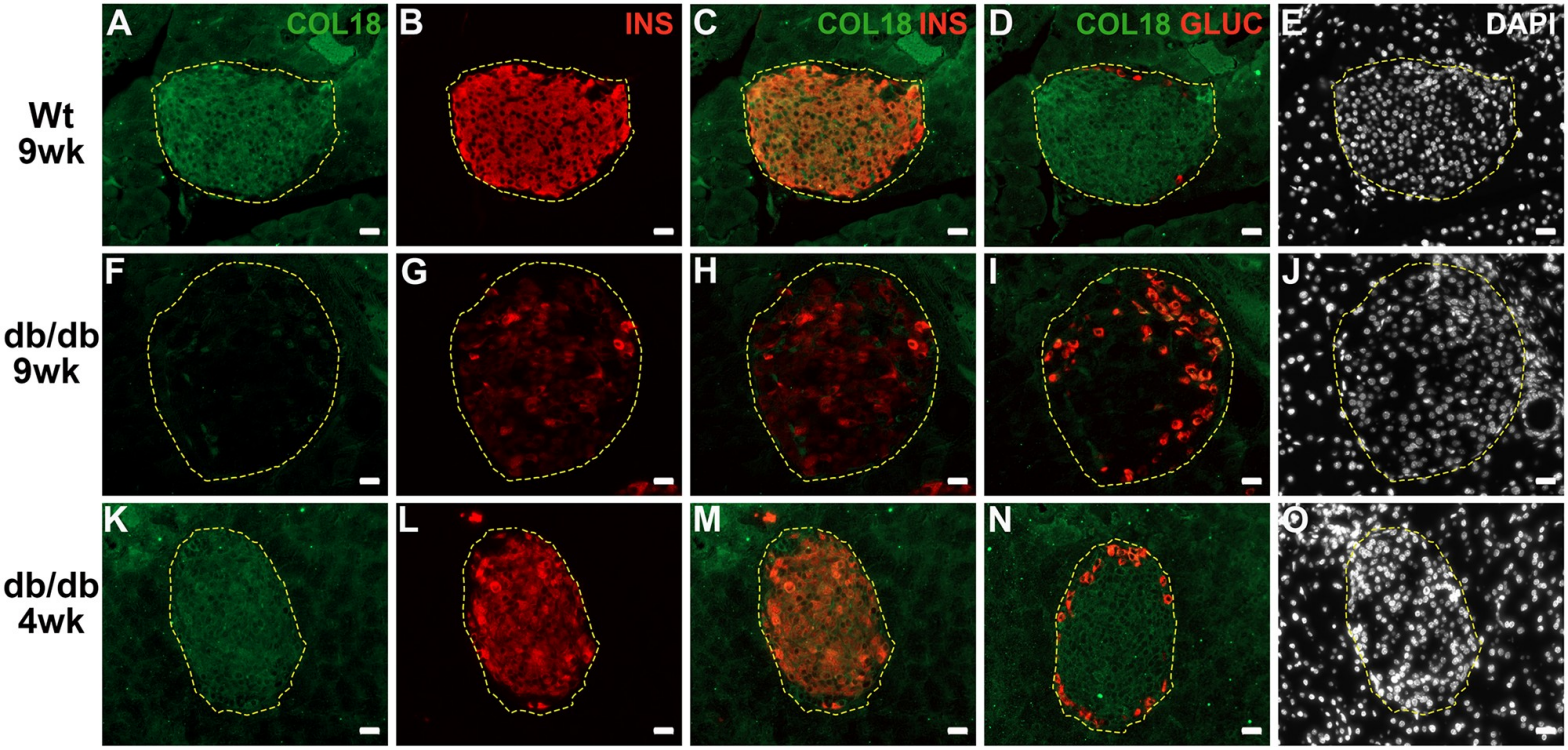
Intra-islet COL18 core protein colocalizes with insulin not glucagon staining in wt pancreas and is progressively lost in db/db pancreas. Immunofluorescence staining of (**A, F, K**,) COL18, (**B, G, L**) insulin (INS), (**C, H, M**) insulin and COL18 merged, (**D, I, N**) glucagon (GLUC) and COL18 merged in 9 wk wt (**A-D**), 9 wk db/db (**F-I**) and 4 wk db/db (**K-N**) pancreas. (**E, J, O**) Nuclei were stained with DAPI. Scale bar = 20 μm.

Firstly we investigated whether like T1D [15, 16], T2D development in db/db mice also correlated with a deterioration in beta cell HS. We extended the analysis to HSPG core proteins, based on (i) the impact of ER stress on global translation/protein synthesis and (ii) the diminished levels of intra-islet HSPGs in T1D human pancreases [16]. Strong immunohistochemical staining for HSPG core proteins (COL18, SDC1 and CD44) and HS was observed in the islets of lean control (wt and db/+) males at 6 weeks of age; in contrast, the islet area stained for HSPGs and HS was significantly decreased in male db/db islets by as early as 3–4 weeks (Fig 1A–1E and S1 File). Thereafter, the area of COL18, SDC1 and CD44 staining in db/db islets declined to 38.8–62.4% of controls by 5 weeks and 18.7–47.2% at 6–9 weeks (Fig 1B–1D and S1 File). Colocalization studies using immunofluorescence staining confirmed that COL18 in wt pancreas (Fig 2A–2D) and 4 wk db/db pancreas (Fig 2K–2N) was restricted to islet beta cells and not alpha cells. A striking loss of COL18 in db/db beta cells was observed in 9 wk db/db islets with scattered insulin-positive beta cells that were COL18-negative (Fig 2G and 2H). The % HS+ve islet area in db/db islets was significantly decreased to 48.1% and 25.7% of lean control islets (wt) at 4 weeks and 9 weeks of age, respectively (Fig 1E and S1 File). Unlike the early decline in % HSPG+ve and HS+ve islet area observed in young db/db islets, the decline observed in lean control islets at 16 weeks and/or 20 weeks may be due to an intrinsic slow core protein turnover [54] or possibly to age-related effects [55]. Compared to lean (wt and db/+) controls, male db/db mice were obese (1.3-fold heavier than corresponding lean controls) and hyperglycemic (db/db, 19.63 ± 2.54 mmol/l vs lean controls, 8.87 ± 0.49 mmol/l) by 6 weeks (S2 Fig). Overall, compared to lean controls, HSPG core proteins and HS were dramatically reduced in the islets of db/db mice, with this perturbation occurring before the onset of hyperglycemia. Similar changes were observed in female db/db islets *in situ* (S3 Fig and S2 File). These findings raised the possibility that the intra-islet levels of HSPG core proteins, and indirectly HS, in db/db beta cells could be impacted by ER stress.

### UPR gene expression is upregulated in db/db islets

**Fig 3:**
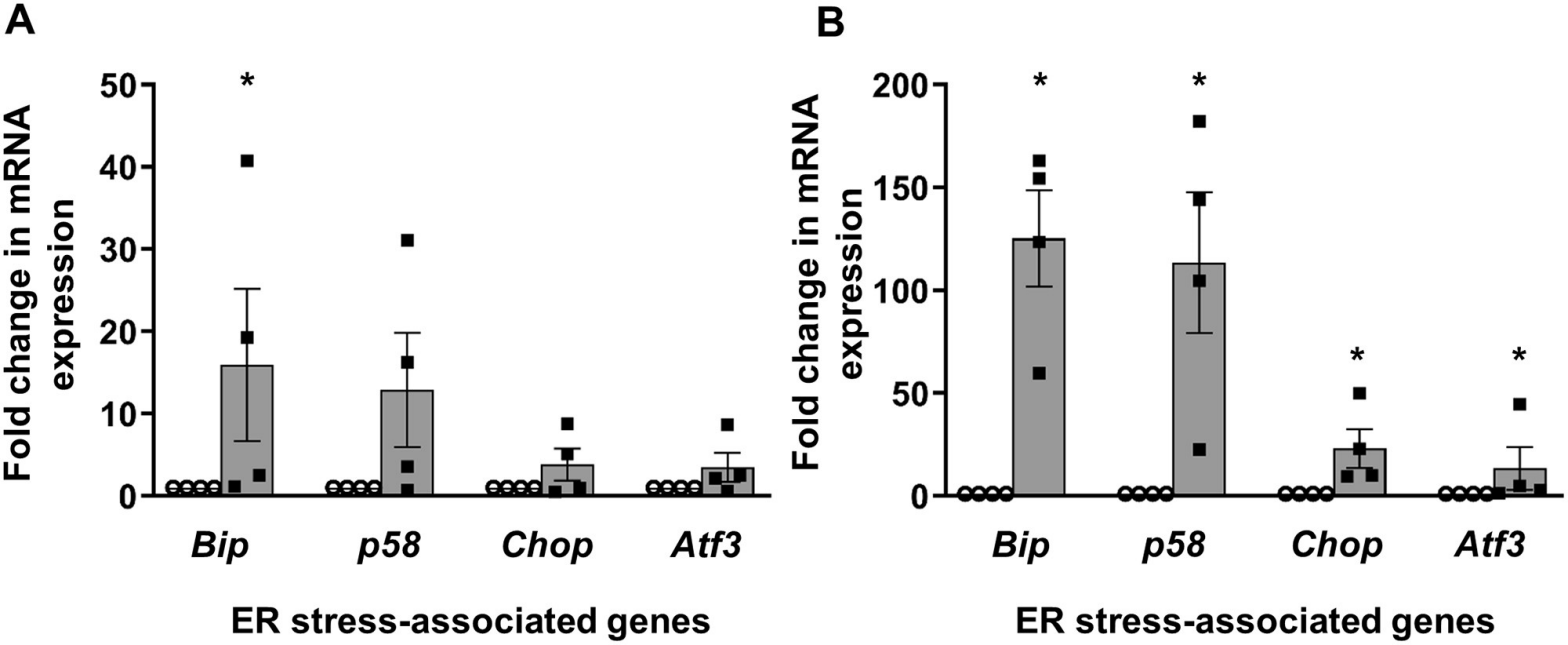
ER stress markers in the islets of male db/db mice at different ages. Relative transcript levels for ER stress genes (*Bip*, *p58*, *Chop* and *Atf3*) in islets of (**A**) young (4.0–4.4 week) lean controls (db/+; open bars) and normoglycemic db/db mice (shaded bars) and (**B**) 5–8 week lean controls (wt; open bars/~baseline) and db/db mice (hyperglycemic; shaded bars). The values were normalized to the house-keeping gene *Gapdh*. The data represent mean ± SEM for 4 independent experiments (120–743 islets/group/experiment). *p<0.05, Mann-Whitney test.

Investigating the relationship between ER stress and levels of intracellular HSPGs and HS in db/db and lean (wt or db/+) beta cells, we found that db/db islets from young donors (4.0–4.4 weeks) showed a significant 15.9-fold upregulation in the early adaptive ER stress-associated gene *Bip* (*p*<0.05). We also observed trends for increased UPR transcripts for *p58* (12.9-fold) as well as *Chop* (3.8-fold) and *Atf* (3.5-fold), i.e., deleterious UPR genes (Fig 3A and S3 File). The mRNA levels for *Bip*, *p58*, *Chop* and *Atf3* were significantly enhanced 125-fold, 113-fold, 23-fold and 13.4-fold, respectively, in islets from older hyperglycemic db/db mice (5–8 weeks; Fig 3B and S3 File). For both age groups we found variable expression of each UPR transcript which we attribute to biological variability, including possible differences in the onset of ER stress between individual young donor mice, variable levels of ER stress in older donors and potentially variability in ER stress gene expression amongst islets from the same donor. The expression of adaptive UPR genes in db/db islets, particularly from older donors, remained selectively increased compared to pro-apoptotic UPR genes. These data suggest a possible mechanism responsible for the loss of intra-islet HSPG core proteins in db/db islets.

### Loss of intra-islet HSPG core proteins and HS in Ins2^WT/C96Y^ mice

**Fig 4:**
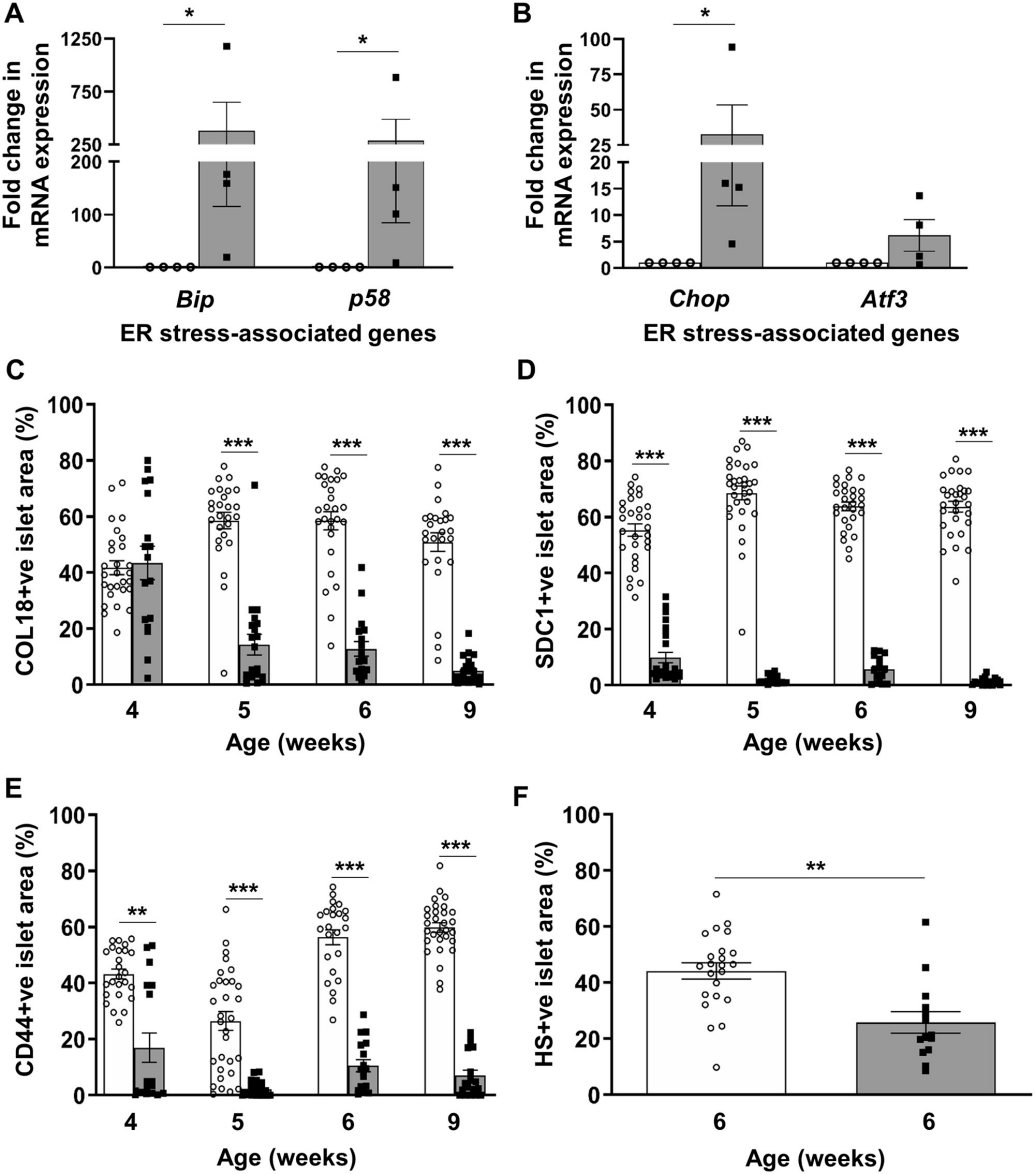
ER stress, intra-islet HSPG core protein and HS expression in Ins2^WT/C96Y^ islets. Fold change in the expression of ER stress-related genes (**A**) *Bip*, *p58* and (**B**) *Chop*, *Atf3* in 5–12 week male Ins2^WT/WT^ control islets (open circles) and Ins2^WT/C96Y^ islets (black squares) was normalized to the house-keeping gene *Gapdh*. Morphometric analysis of immunostained intra-islet HSPG core proteins and HS in Ins2^WT/WT^ (open bars) and Ins2^WT/C96Y^ (shaded bars) pancreases at 4, 5, 6 and 9 weeks (HSPGs) and 6 weeks (HS). Data is presented as the % islet area with positive staining for (**C**) COL18, (**D**) SDC1, (**E**) CD44 and (**F**) HS. Data represent mean ± SEM for (**A-B**) n = 4 independent experiments (250–350 islets/group/experiment) and (**C-F**) n = 3 pancreases/age group with n = 15–31 islets examined/group; each symbol denotes an individual islet. *p<0.05, **p<0.001 and ***p<0.0001, Mann-Whitney test.

We further explored the relationship between ER stress and the loss of HSPG core proteins/HS in beta cells using Ins2^WT/C96Y^ (Akita) mice, an established model of ER stress-mediated diabetes [56]. TaqMan qRT-PCR showed a 287–383 fold increase in adaptive UPR mRNAs (*Bip* (*p*<0.05), *p58* (*p*<0.05); Fig 4A and S4 File) in isolated heterozygous Ins2^WT/C96Y^ islets and a 33 fold increase in the expression of *Chop*, a deleterious UPR gene (*p*<0.05; Fig 4B and S4 File), compared to control Ins2^WT/WT^ islets. We also observed a trend for a 6-fold increase in *Atf3* transcripts but this increase did not reach statistical significance. Immunohistochemistry showed that the COL18+ve islet area in Ins2^WT/C96Y^ islets was reduced to 24.5% of control Ins2^WT/WT^ islets by 5 weeks (*p*<0.0001; Fig 4C, S4 Fig and S4 File). The intra-islet SDC1 and CD44 core protein levels were also significantly reduced at 4–9 weeks (*p*<0.0001; Fig 4D and 4E, S4 Fig and S4 File). In parallel, the HS+ve islet area in Ins2^WT/C96Y^ islets was significantly decreased to 58.4% of controls at 6 weeks (*p*<0.001; Fig 4F, S4 Fig and S4 File). Ins2^WT/C96Y^ mice therefore demonstrated a significant loss of intra-islet HSPG core proteins and HS, correlating with acute ER stress and early diabetes development.

### Intracellular HSPG core proteins, HS and HPSE in MIN6 cells ± ER stress

**Fig 5:**
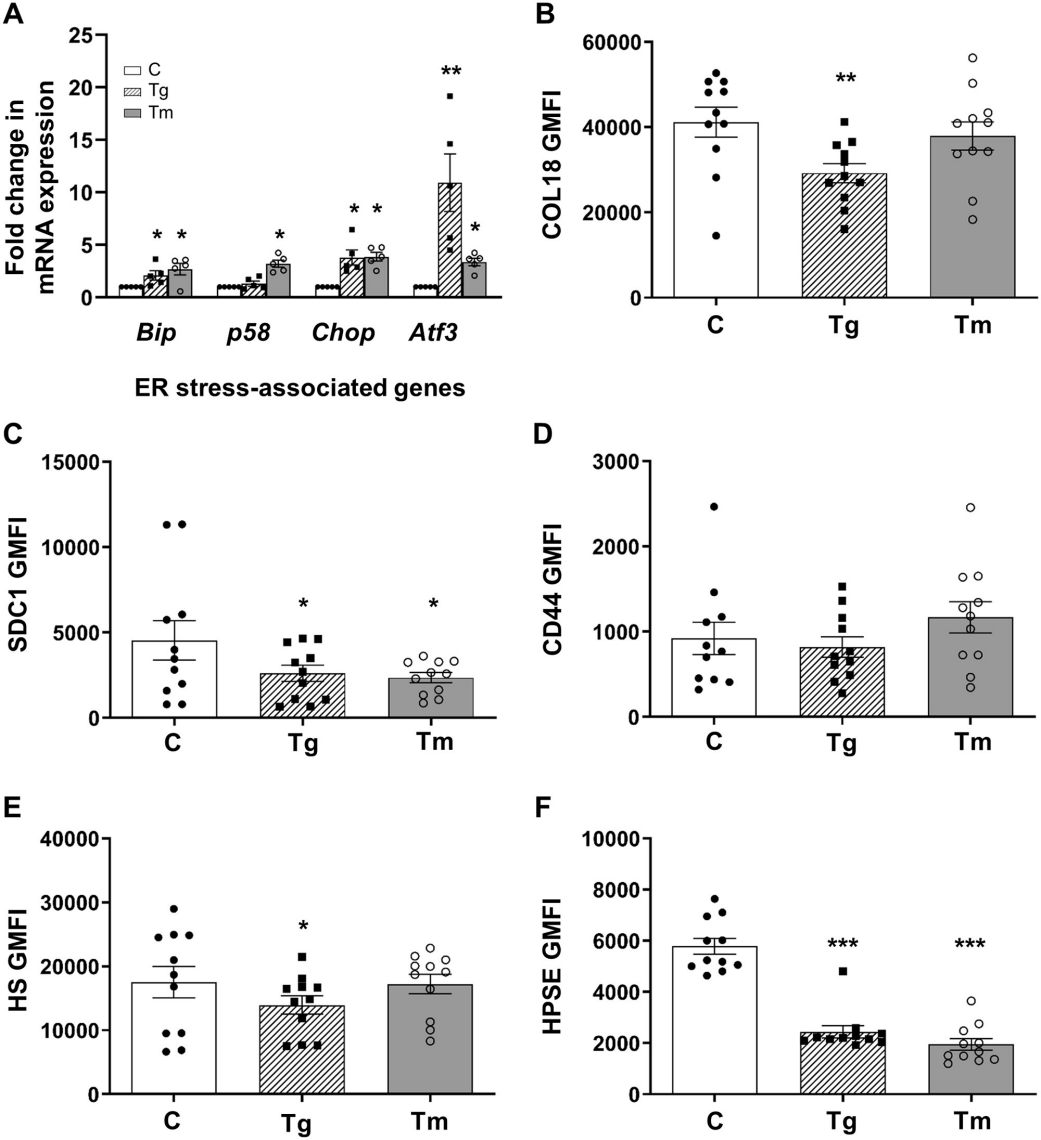
Intracellular levels of HSPG core proteins, HS and HPSE are decreased in ER stressed MIN6 cells. (**A**) MIN6 cells were cultured with 6 mM glucose (open bars), 50 nM thapsigargin (striped bars) or 2 μM tunicamycin (grey bars) for 20–24 hours. Transcript levels for *Bip*, *p58*, *Chop* and *Atf3* were normalized to the house-keeping gene *Ube2d1* and were quantified as a fold change compared to 6 mM control mRNA levels which were assigned a value of 1. (**B-F**) MIN6 cells were cultured for 3 days with 6 mM glucose-DMEM (control cells, C), 50 nM thapsigargin (Tg) or 2 μM tunicamycin (Tm) and intracellular HSPG core protein (**B**) COL18, (**C**) SDC1 and (**D**) CD44, (**E**) HS and (**F**) HPSE levels were assessed by flow cytometry and expressed as GMFI. Data represent mean ± SEM for (**A**) n = 5 independent experiments and (**B-F**) n = 11 experiments. *p<0.05, **p<0.01 and ***p<0.001, One-way ANOVA with Fischer’s unprotected LSD test.

We next used MIN6 beta cells [44] to experimentally induce ER stress and to monitor beta cell HSPGs and HS levels. Firstly, we showed by flow cytometry that freshly harvested MIN6 cells resembled primary beta cells [15, 27] with strong expression of intracellular COL18 core protein (S5A Fig), HS (S5B Fig) and HPSE (S5C Fig) and low cell surface levels (S5 Fig). Thapsigargin treatment of MIN6 cells upregulated the UPR transcripts *Bip* (*p*<0.05), *Chop (p*<0.05) and *Atf3 (p*<0.01) and treatment with tunicamycin significantly increased the expression of *Bip*, *p58*, *Chop* and *Atf3* mRNAs (*p*<0.05), confirming the induction of ER stress (Fig 5A and S5 File). Extending the duration of *in vitro* treatment to 3 days, thapsigargin significantly decreased intracellular HPSG core proteins (COL18, *p*<0.01; SDC1, *p*<0.05) and HS (*p*<0.05) to 57.5–70.9% and 79.8% of controls (Fig 5B, 5C and 5E and S5 File); in parallel, HPSE levels declined to 42.2% of controls (*p*<0.001; Fig 5F and S5 File). Similarly, tunicamycin treatment also lowered intracellular SDC1 (*p*<0.05) and HPSE (*p*<0.001) levels to 52.0% and 33.6% of control levels (Fig 5C and 5F and S5 File). Neither treatment reduced intracellular CD44 core protein (Fig 5D and S5 File). However, the ER stress response induced pharmacologically in MIN6 cells was much less severe than that observed endogenously in native beta cells *in vivo* (Figs 3A, 3B, 4A and 4B). Accordingly, the reduced levels of a subset of intracellular HPSG core proteins and HS were less pronounced than those observed in mouse models of ER stress (Figs 3 and 4). Importantly, thapsigargin-treated MIN6 cells demonstrated a loss of both intracellular HPSE and HS, providing evidence that ER stress-induced HS loss was not due to HS degradation.

### Intracellular HSPGs, HS and HPSE are reduced in isolated db/db beta cells

**Fig 6:**
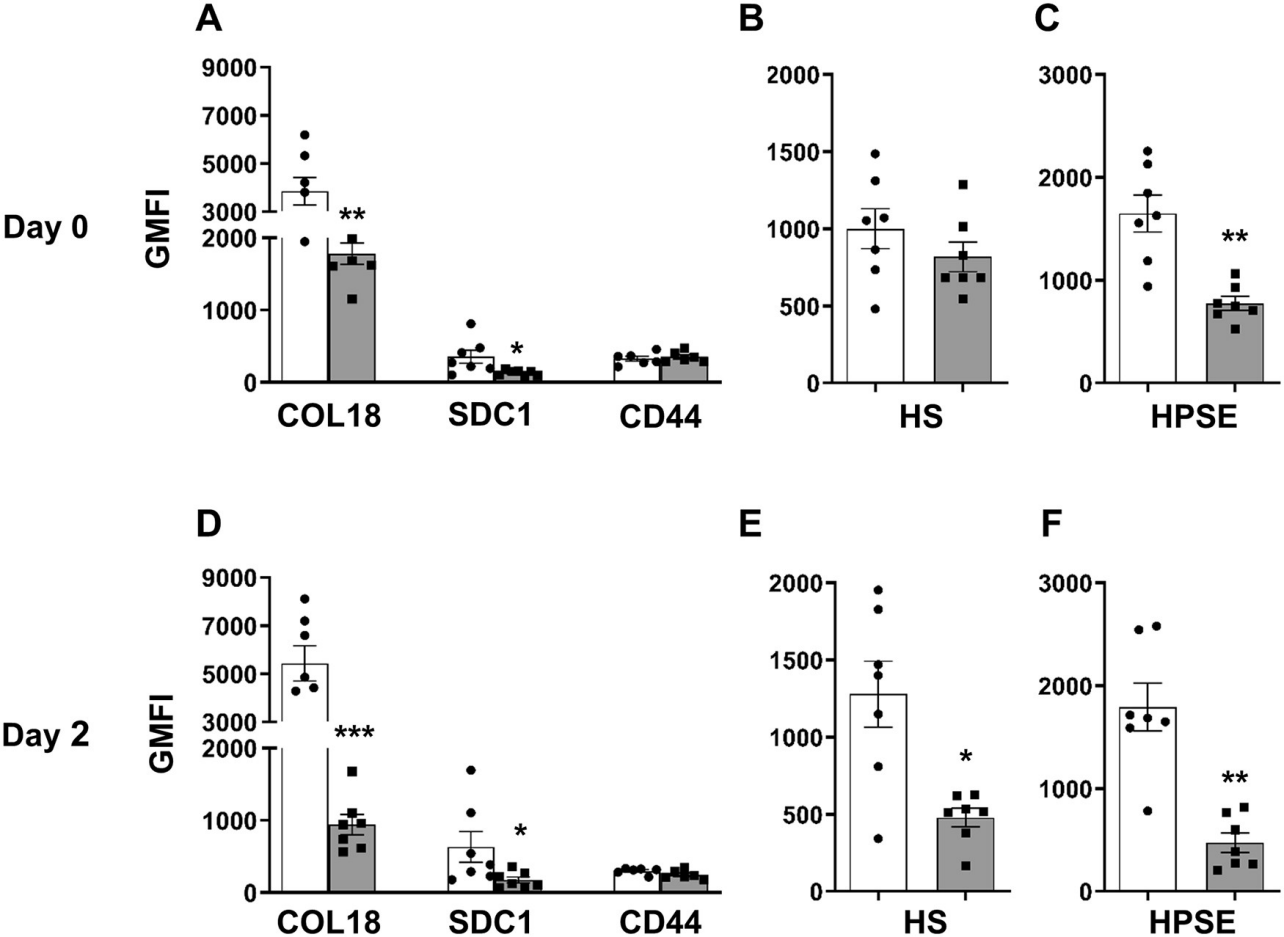
Intracellular expression of HSPGs, HS and HPSE in wt and db/db isolated islet cells. Intracellular levels of (**A, D**) HSPG core proteins COL18, SDC1 and CD44, (**B, E**) HS and (**C, F**) HPSE in wt (open bars) and db/db (shaded bars) isolated islet cells on day 0 and after culture for 2 days (day 2). Data show GMFI ± SEM, n = 7 experiments/group with n = 2–4 male donors/experiment. *p<0.05, **p<0.01 and ***p<0.001, compared to corresponding control wt cells, Mann-Whitney test.

Using flow cytometry, we found that HSPG core proteins, HS and HPSE are 3.2–34.2 fold, 8.0 fold and 12.6 fold more highly expressed intracellularly than at the cell surface in freshly isolated wt beta cells, respectively (Fig 6, S6 Fig, S4 Table and S6 File). Furthermore, in agreement with immunohistochemical studies of host pancreas (Fig 1), we demonstrated significantly reduced intracellular expression of HSPG core proteins (COL18, SDC1) in db/db beta cells to 36–47% of wt cells (Fig 6A and 6D and S6 File). In addition, a rapid deterioration of COL18, HS and HPSE to 53%, 59% and 61% of day 0 levels, respectively, was observed inside db/db beta cells but not wt beta cells during culture for 2 days (Fig 6B and 6E, S4 Table and S6 File). Like ER-stressed MIN6 cells (Fig 5F), intracellular HPSE was significantly reduced in db/db beta cells to 26% of wt controls (Fig 6C and 6F, S4 Table and S6 File), indicating that the decline in db/db beta cell HS during culture was HPSE-independent. We next investigated whether the major loss of HS in cultured db/db beta cells contributes to their failure.

### HS replacement improves db/db beta cell viability and protects against ROS-induced cell death

**Fig 7:**
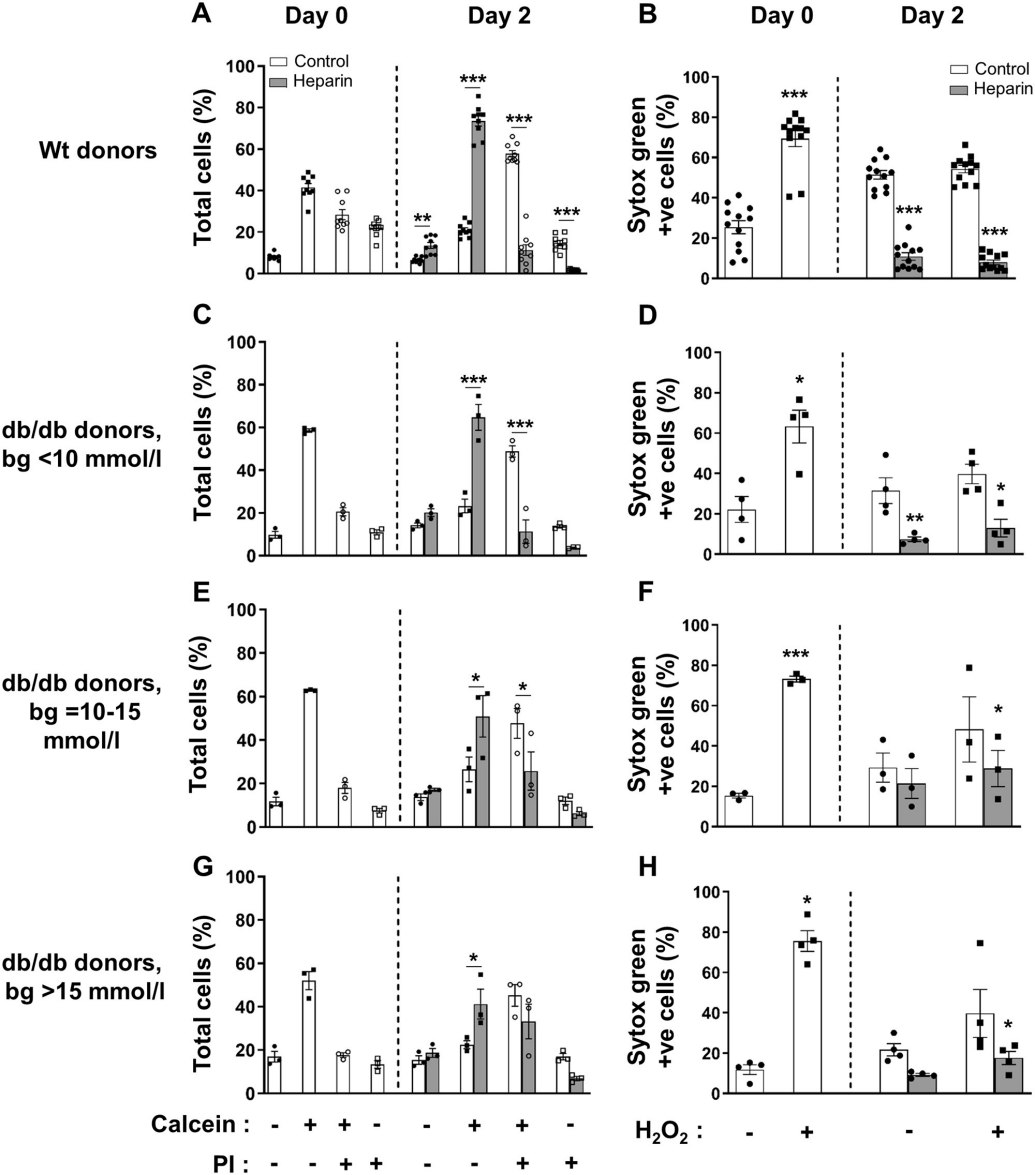
HS replacement protects db/db beta cells from dying in culture and from acute hydrogen peroxide-induced damage. The viability of (**A**) wt and (**C, E, G**) db/db beta cells was analyzed by flow cytometry using the fluorescent dyes Calcein (Cal) and PI after culture for 2 days without (open bars) or with (shaded bars) the highly sulfated HS analogue heparin (50 μg/ml). Cell death/damage due to acute treatment with hydrogen peroxide was measured by uptake of the fluorescent dye Sytox Green. Donors were (**A, B**) wt; (**C, D**) normoglycemic db/db (bg<10 mmol/l); (**E, F**) mildly hyperglycemic db/db (bg = 10–15 mmol/l) and (**G, H**) severely hyperglycemic db/db (bg>15 mmol/l). Beta cells were identified as viable (Cal^+^PI^-^), damaged (Cal^+^PI^+^) or dead (Cal^-^PI^+^) and expressed as % total cells; alternatively, damaged/dead cells were identified as Sytox Green+ve. Data represent mean ± SEM for n = 3–11 experiments/group; n = 2–4 male donors/experiment. *p<0.05, **p<0.01 and ***p<0.0001, ANOVA with Fisher’s unprotected LSD post-test.

We tested whether db/db beta cells, like normal mouse and human beta cells [15, 16], can be rescued by HS replacement during culture for 2 days with heparin (a highly sulfated HS analogue; 50 μg/ml; S7 Fig). Initially, the survival of freshly isolated wt beta cells (Cal^+^PI^-^; 41.49 ± 1.92%) was lower than for db/db beta cells from normoglycemic donors (58.60 ± 0.78%). After culture, the viability of wt beta cells and db/db beta cells (Cal^+^PI^-^) declined to 50.5% and 39.6% of Day 0 levels, respectively; similarly, the viability of beta cells from other db/db donors (with blood glucose levels of 10–15 mmol/l and > 15 mmol/l) was reduced to 42.2%-43.2% of Day 0 levels (S7 File). Culture of wt beta cells with heparin (which replaces highly sulfated HS that is selectively localized in beta cells [31] and lost during the isolation process [15]) significantly increased the live (Cal^+^PI^-^) population of beta cells by 3.5-fold and reduced damaged Cal^+^PI^+^ beta cells and Cal^-^PI^+^ dead cells to 10.5–19.6% (*p*<0.001) of untreated wt controls (Fig 7A and S7 File). Similarly, heparin significantly increased the survival of beta cells from male normoglycemic db/db mice (bg<10 mmol/l) by 2.8-fold (*p*<0.001), and from mildly hyperglycemic db/db donors (bg = 10–15 mmol/l) and hyperglycemic donors (bg>15 mmol/l) by 1.9-fold (*p*<0.05; Fig 7C, 7E and 7G and S7 File). PI-88, another highly sulfated HS mimetic and HS replacer, preserved the survival of wt beta cells as previously reported [15] but was less effective than heparin in protecting the viability of db/db beta cells (S8 Fig). HS replacement using heparin therefore vastly improved the survival of db/db beta cells.

In parallel, db/db beta cells were analyzed for susceptibility to H_2_O_2_-induced damage by Sytox Green staining (S9 Fig). Freshly isolated (Day 0) wt beta cells (Fig 7B and S7 File) and beta cells from normoglycemic (Fig 7D and S7 File), mildly hyperglycemic (Fig 7F and S7 File) and severely hyperglycemic (Fig 7H and S7 File) db/db mice demonstrated a significant 2.7 (*p*<0.0001), 2.9 (*p*<0.05), 4.8 (*p*<0.0001) and 6.4-fold (*p*<0.05) increase in cell death following acute H_2_O_2_ treatment. Hyperglycemic db/db beta cells were therefore more highly sensitive to acute oxidative damage than normoglycemic db/db beta cells or wt controls. This vulnerability was noticeably mitigated after 2 days of culture without heparin in all groups, probably reflecting protection of beta cell viability due to the induced expression of conventional oxidative stress-related genes e.g., ROS-scavenging enzymes [3, 57]. In the presence of heparin, wt (Fig 7B and S7 File) and normoglycemic db/db (Fig 7D and S7 File) beta cells significantly decreased Sytox Green+ve beta cell damage/death to 21.1% (*p*<0.001) and 23.4% (*p*<0.01) of untreated controls, resembling viability assessment using Calcein/PI staining (Fig 7A and 7C and S7 File). Strikingly, heparin treatment also protected wt and db/db beta cells from H_2_O_2_-induced death with only ~8% Sytox Green+ve damaged/dead cells (versus 54% without heparin; Fig 7B; *p*<0.001; S7 File) and ~12–30% Sytox Green+ve cells (versus 39–48% without heparin; Fig 7D, 7F and 7H; *p*<0.05; S7 File), respectively. These data illustrate that HS replacement *in vitro* using heparin (i) renders db/db beta cells resistant to acute oxidative stress and (ii) represents a more efficient mechanism than other endogenous pathways induced/enhanced during culture in response to oxidative stress.

### TUDCA treatment of db/db mice improves glycemia and beta cell HSPG/HS levels

**Fig 8:**
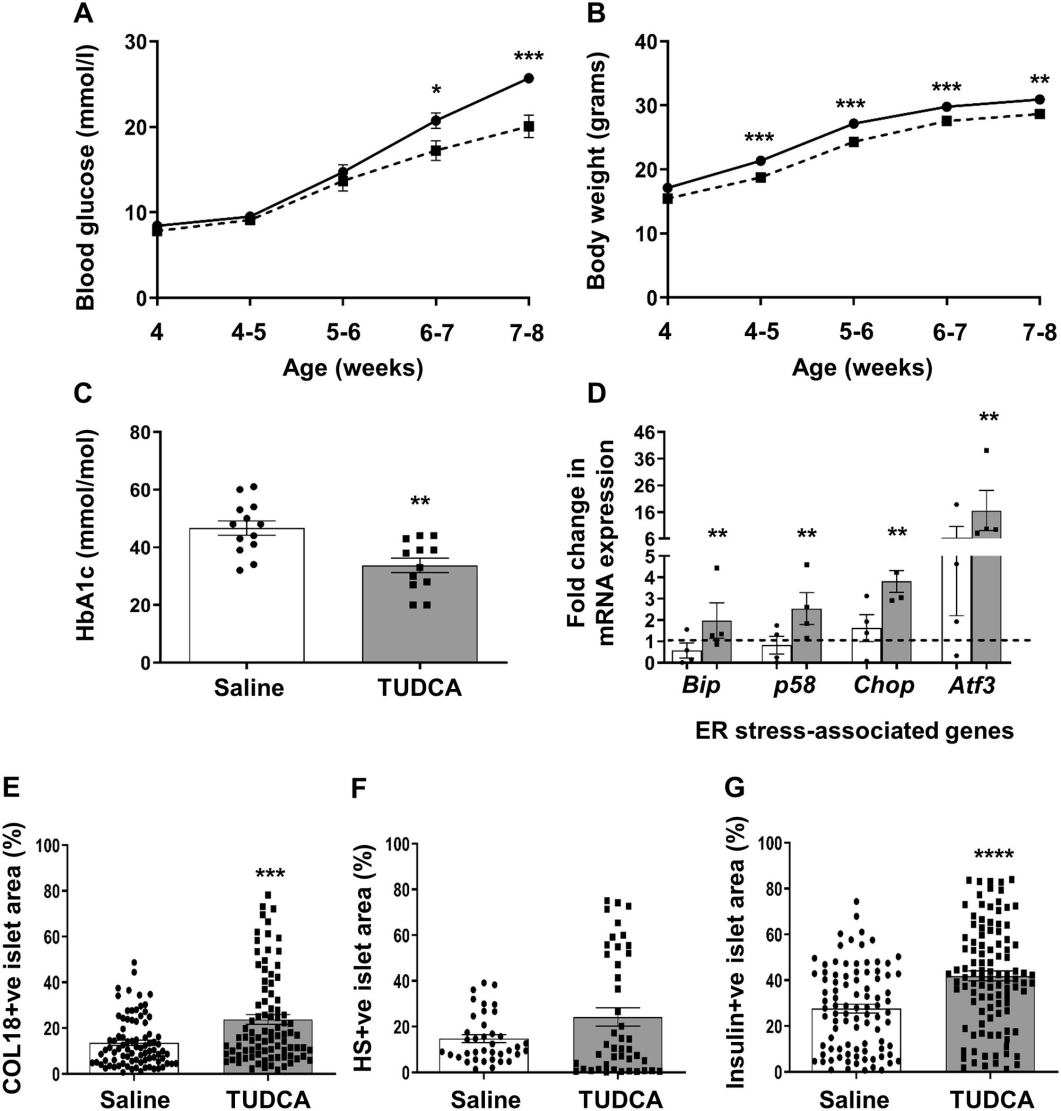
Effect of TUDCA treatment of db/db mice on glycemic control, ER stress-related gene expression and intra-islet COL18 and HS. 4 week old male db/db mice were treated with saline (black line) or TUDCA (150 mg/kg/day; broken line) i.p. for 28 days. (**A**) The non-fasting blood glucose levels and (**B**) body weight were monitored 3x/week. (**C**) HbA1c levels were measured at termination of treatment. (**D**) ER stress-related gene transcripts were analyzed in islets isolated from saline-treated (open bars) and TUDCA-treated (shaded bars) db/db mice. Fold-change refers to mRNA expression relative to gene expression in wt kidney (broken line) which was assigned a value of 1 and normalized to the house-keeping gene *Gapdh*. The *in situ* expression of (**E**) HSPG core protein (COL18), (**F**) HS and (**G**) insulin was determined by immunohistochemistry and evaluated as % islet area stained. (**A-B**) Data represent mean weekly measurements ± SEM with n = 12–14 male mice/group, n = 3 measurements/mouse; (**C**) mean ± SEM for n = 12–13 mice/group; (**D**) mean ± SEM, each data point represents an independent experiment, n = 4 mice/group (100–140 islets/donor); (**E-G**) n = 4–9 pancreases/group with n = 86–88 (COL18), n = 38–44 (HS) and n = 92–105 (insulin) islets examined/group. *p<0.05, **p<0.01, ***p<0.001, and ****p<0.0001, Mann-Whitney test.

Our studies have revealed that ER-stress in db/db islets correlates with a rapid decline in islet/beta cell HSPGs and HS. Similarly, the pharmacological induction of ER stress in MIN6 cells results in loss of intracellular HSPGs and HS. Against this background we interrogated the relationship between ER stress, T2D and loss of beta cell HSPG/HS *in vivo* by treating db/db males from 4 weeks of age with the ER chemical chaperone TUDCA. TUDCA-treated male db/db mice showed impaired progression of T2D (Fig 8A and S8 File), decreased body weight (Fig 8B and S8 File) and improved control of glycemia (Fig 8C and S8 File). Also at termination, isolated db/db islets showed a significant 3.1–3.5 fold (*p*<0.01) increase in the expression of adaptive UPR genes (*Bip* and *p58*) and a 2.3–2.6 fold (*p*<0.01) increase in the expression of pro-apoptotic UPR genes (*Chop* and *Atf3)*, compared to saline controls (Fig 8D and S8 File). These findings suggest that TUDCA elevated the UPR to enhance beta cell compensation, maintain beta cell function and thus improve glycemia.

**Fig 9:**
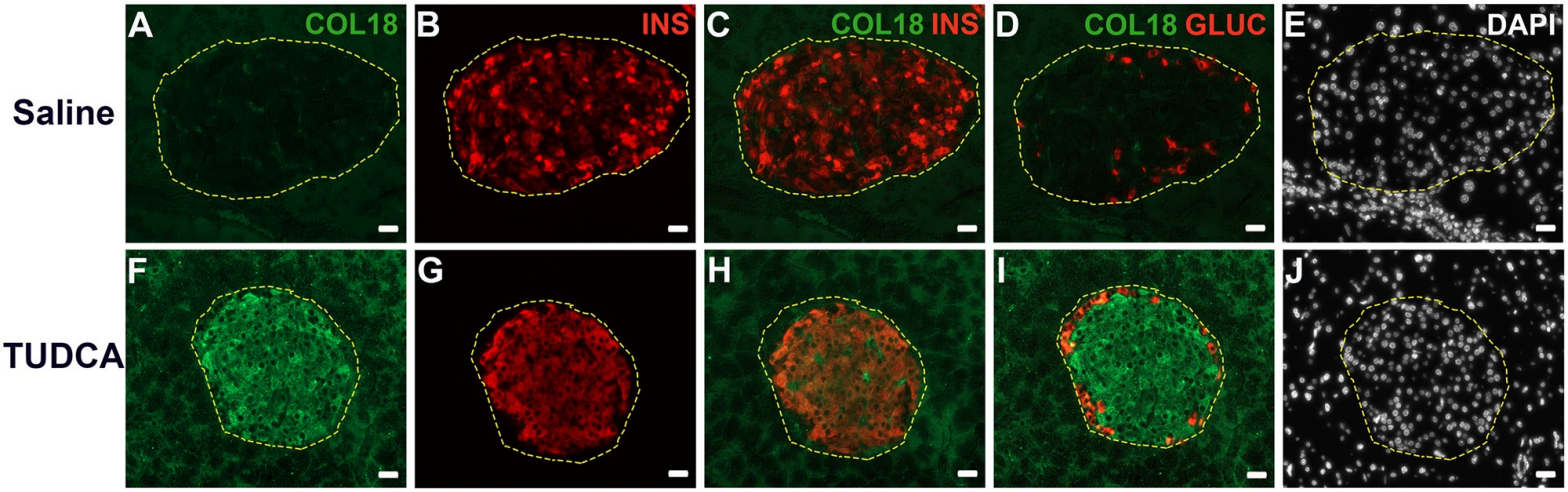
TUDCA treatment restores the colocalization of intra-islet COL18 core protein and insulin in db/db pancreas. Immunofluorescence staining of (**A, F**) COL18, (**B, G**) insulin (INS), (**C, H**) insulin and COL18 merged, (**D, I**) glucagon (GLUC) and COL18 merged in (**A-D**) saline-treated control and (**F-I**) TUDCA-treated db/db pancreases. (**E, J**) DAPI was used to identify nuclei. Scale bar = 20 μm.

We next assessed whether relieving ER stress influenced the intra-islet expression of HSPG core protein COL18 and HS. Immunohistochemical staining of host pancreas showed that the COL18+ve islet area was significantly increased by 1.7-fold in the islets of TUDCA-treated db/db mice (23.79 ± 2.12%), compared to controls (13.65 ± 1.17%, *p*<0.001; Fig 8E and S8 File). Compared to saline-treated controls which demonstrated loss of intra-islet COL18 and only scattered insulin-positive beta cells (Fig 9A and 9B), TUDCA treatment resulted in widespread intra-islet COL18 staining (Fig 9F) and uniform staining of intra-islet insulin (Fig 9G) which colocalized with COL18 (Fig 9H). The HS+ve islet area also increased 1.6-fold (24.22 ± 3.95% versus 14.80 ± 1.72% in saline controls; Fig 8F and S8 File) and was accompanied by a 1.5-fold increase in the insulin+ve islet area (*p*<0.0001; Fig 8G and S8 File). The striking improvement in db/db beta cell HS therefore correlated with a significant increase in the expression of HSPG core proteins and an enhanced adaptive UPR to help compensate for ER stress and counter pro-apoptotic UPR effects.

## Discussion

We report here that beta cell HSPG core proteins and highly sulfated HS are both significantly depleted in prediabetic 3–5 week db/db mice. These events correlated with the preferential increase in *Bip* and *p58* (*Dnajc3*) mRNAs, representing markers of the UPR, and preceded both overt hyperglycemia and significant weight gain from 6 weeks of age. Similarly, we demonstrated by quantitative immunohistochemical analyses, a dramatic decline in beta cell HSPGs and HS in the islets of young 4–5 week Ins2^WT/C96Y^ mice, a transgenic model of ER-stressed beta cells [56]. Flow cytometry studies revealed that, *in vitro*, acute pharmacological induction of ER stress in MIN6 cells by thapsigargin and tunicamycin, significantly reduced intracellular levels of the HSPG core proteins COL18 and SDC1. This outcome is attributed to a general decrease in protein expression resulting from the activation of UPR genes. Furthermore, the impact of ER stress is likely to be influenced by the rate of protein turnover, which may vary for different HSPGs. Ultimately a decline in HSPG core proteins would have the downstream effect of decreasing intracellular HS. We also observed a parallel decline in intracellular HPSE (HS-degrading endoglycosidase) in both ER stressed MIN6 cells and db/db beta cells, which precluded HS degradation as the mechanism responsible for HS loss.

**Fig 10:**
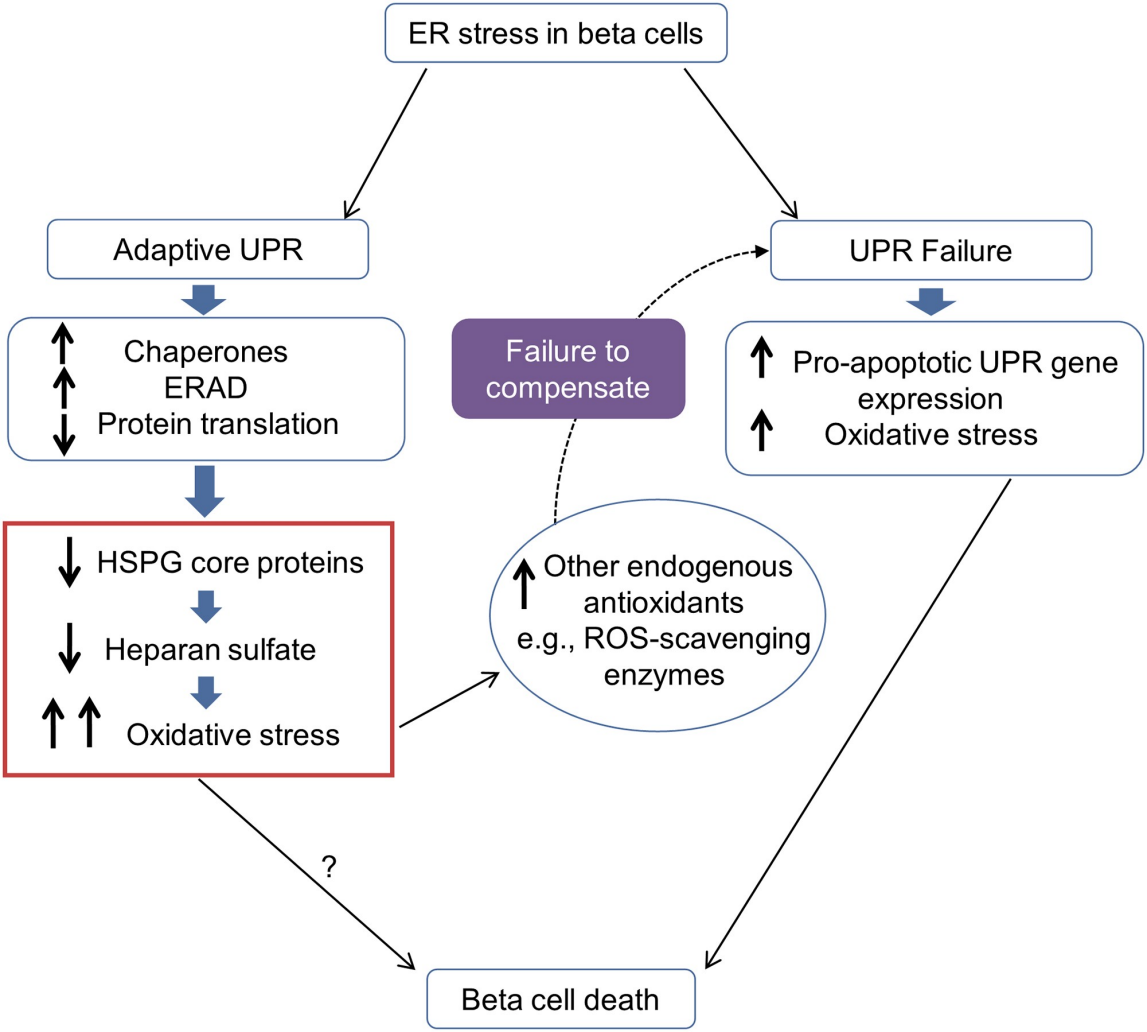
The proposed model: ER stress inhibits the synthesis of HSPG core proteins and HS in T2D-prone beta cells. In pre-T2D pancreatic beta cells, ER stress initiates the unfolded protein response (UPR) which acts to alleviate the stress by increasing molecular chaperones for protein folding, increasing ERAD and reducing the translation of mRNAs to a range of “general” proteins. As a consequence, HSPG core protein synthesis is impaired, which in turn severely reduces the synthesis of HS, a constitutive non-enzymatic antioxidant in beta cells. Depletion of beta cell HS increases intracellular ROS and elevates oxidative stress. Oxidative stress induces the expression of secondary antioxidant enzymes to help maintain beta cell homeostasis [57]. Failure to compensate for this stress results in UPR failure, the preferential expression of apoptotic genes and beta cell apoptosis/death. ERAD, ER-associated degradation; T2D, Type 2 diabetes; HS, heparan sulfate; HSPG, heparan sulfate proteoglycan; ROS, reactive oxygen species.

We have previously reported that beta cell HS is depleted during the isolation of normal mouse and human islets *in vitro*, and renders isolated beta cells especially susceptible to ROS-induced death [15, 16, 27]. Furthermore, HS replacement using HS mimetics reverses this heightened vulnerability and preserves beta cell viability [15, 16]. From our current study, we propose that the significant decrease in intracellular HSPG core proteins (COL18 and SDC1) and HPSE in freshly isolated db/db beta cells, compared to wt beta cells, is due to ER stress in the donor db/db mice. While there was a trend for a greater reduction in intracellular HS in db/db beta cells *in vitro* (Fig 6), we reason that any pre-existing impact of ER stress was masked by the acute loss of HS during the isolation of wt beta cells used for comparison. This *in vitro* manipulation-induced loss of HS is most likely due to the depolymerization of HS by excessive ROS generated during the islet isolation procedure [15, 27, 58, 59]. Paradoxically, freshly isolated db/db beta cells initially demonstrated better viability than wt beta cells. We reason that in contrast to wt islets, islets derived from db/db donors have higher endogenous levels of antioxidant enzymes [3, 57, 60], induced *in vivo* in response to endogenous oxidative stress associated with ER stress-induced loss of HSPG core proteins/HS. We propose that this induced antioxidant response compensates for loss of HS (Fig 10) in the initial post-isolation period.

We observed a continued decline in HSPGs (COL18 and SDC1), HS and HPSE during the culture of db/db beta cells, a finding not observed in wt beta cells and consistent with the perpetuation of endogenous ER stress *in vitro*. Interestingly, treatment of both wt and db/db beta cells with the HS replacer heparin significantly improved the survival of beta cells (Cal^+^PI^-^ cells) from normoglycemic, mildly hyperglycemic and severely hyperglycemic db/db donors and protected them from hydrogen peroxide-induced death. On this basis, we propose that the loss of HS from beta cells of prediabetic db/db mice *in vivo* exacerbates oxidative stress. Surprisingly, another highly sulfated HS mimetic, PI-88, acted as a HS replacer and protected wt beta cells but not db/db beta cells (S8 Fig). It is possible that different heparin-binding proteins [25] may be used in the uptake and/or intracellular distribution of heparin and PI-88 in db/db beta cells and could lead to a difference in susceptibility to ER stress effects.

Consistent with previous reports of genetically obese mice treated with chemical chaperones [38, 61], treatment of db/db mice with the ER chaperone TUDCA significantly reduced non-fasting blood glucose levels, body weight and HbA1c levels. In parallel, we observed a significant increase in the intra-islet distribution of COL18+ve which was accompanied by a striking increase in highly sulfated HS. In addition, we observed a significantly higher insulin-positive islet area, a finding consistent with improved islet insulin content after TUDCA treatment in a T1D mouse model [62]. Our findings therefore revealed a positive impact of TUDCA on beta cells and glycemic control. Paradoxically, TUDCA treatment of db/db mice, unlike a previous report of TUDCA-treated db/db mice [61], failed to mitigate the expression of apoptotic UPR mRNAs. The difference between the studies could be due to differences in the age of the mice at commencement of treatment, duration of treatment, tissues analyzed and to the lower dosage regimen of TUDCA in our study. Additionally, the activation of pro-apoptotic pathways could be due to ER stress-independent activation of c-Jun N-terminal kinase (JNK) by oxidative stress or lipotoxicity [63–65]. Increased *Chop* expression has previously been observed during beta cell adaptation in other obese rodent models (Zucker fatty rats, HFD mice), suggesting that a balance between *Chop* expression and adaptive UPR is important for maintaining beta cell function and survival [18, 66]. Since TUDCA treatment of db/db mice improved glycemic control, we propose that the accompanying preferential increase in the expression of adaptive UPR genes compared to deleterious pro-apoptotic genes (*Chop*, *Atf3*) relieved ER stress, prolonged beta cell compensation and promoted recovery of beta cell HSPGs/HS.

Together our *in vitro* and *in vivo* intervention studies highlight a previously unrecognized inter-relationship between HS/HSPGs, oxidative stress and ER stress. While ER stress and oxidative stress have long been recognized as accomplices in beta cell failure in T2D, mechanistic links have remained elusive. Based on our studies we propose a novel pathway which bridges ER stress and oxidative stress in T2D. Here, we postulate that ER stress, prior to the onset of T2D, results in a UPR-induced decline in the synthesis of HSPG core proteins within the ER. Consequently, HSPG core proteins fail to be transferred to the Golgi compartment, impairing or blocking HS synthesis because HSPG core proteins are an essential scaffold for HS assembly [20, 21, 23]. HS deficiency in the beta cells results in increased intracellular ROS and thus, debilitating oxidative stress (Fig 10). This new understanding provides a rational basis for the previously observed upregulation of antioxidant enzyme transcripts [60] and the therapeutic effect of chemical and physiological antioxidants on beta cell mass and/or function in db/db mice [60, 67, 68], a strategy that would compensate for the depletion of endogenous beta cell HS. It is possible that glucotoxicity-associated ER stress in the db/db (genetic) model of T2D, resulting in the depletion of ER Ca^2+^, contributes to the decline in beta cell HSPG core proteins [69]. Future studies could investigate whether obesity, lipotoxicity and ER stress induced by a long-term high-fat diet (for 16 weeks) in normal mice [70] also impacts beta cell HSPGs and HS.

T2D and T1D have distinct metabolic and autoimmune aetiologies, respectively, and different mechanisms of beta cell death [65, 71]. We demonstrate here that prediabetic db/db mice, like pre-diabetic NOD mice, display a loss of HS from beta cells. However, we reveal in this study that the HS-depleting pathways are vastly different. In T1D, the primary mechanism is due to HPSE-dependent degradation of HS [15, 16]. In contrast, our data strongly suggest that HS depletion in T2D beta cells is HPSE-independent; instead, our findings are consistent with the inhibition of HS synthesis, a secondary effect of ER stress on the synthesis/maturation of HSPG core proteins. However, since loss of HSPGs [16] and ER stress have been reported in human T1D beta cells [6, 7, 34, 35, 65], we cannot exclude the possibility that ER stress-induced deprivation of HSPG core proteins may function as an ancillary mechanism for HS loss in T1D. Altogether, loss of beta cell HS is a fundamental defect underpinning pre-diabetes and culminates in insurmountable oxidative damage.

In summary, our studies highlight that intracellular HS acts as a critical link between ER stress and oxidative stress in T2D beta cells i.e., ER stress indirectly reduces beta cell HS, which in turn heightens the susceptibility of beta cells to oxidative stress. Based on the reported islet-associated ER stress in obesity-driven human T2D [17], we speculate that loss of beta cell HSPGs/HS is likely to precede beta cell failure in human T2D. We suggest that treatment with a heparin-like HS replacer, possibly combined with a chemical chaperone, could represent a novel therapeutic strategy for rescuing beta cell survival, prolonging beta cell compensation and alleviating oxidative stress in T2D.

## Supporting information

S1 Table Primer/probe set for TaqMan RT-PCR. (DOCX)

S2 Table Antibodies used for flow cytometry. (DOCX)

S3 Table Antibodies used for immunohistochemistry. (DOCX)

S4 Table Cell surface and intracellular expression of HSPG core proteins, HS and HPSE in wild-type and db/db mouse beta cells. (DOCX)

S1 Fig Localization of CD44 core protein in pancreatic islets of wild-type mice. Immunostaining of the same islet in wt (+/+) pancreas (9 weeks of age) shows similar localization of CD44 using (**A**) rat anti-mouse CD44 (IM7) mAb and (**B**) mouse anti-human CD44v3 mAb. Scale bar = 100 μm.
(TIF)

S2 Fig Blood glucose and body weight of male lean controls and db/db mice at 3–20 weeks of age. (**A**) Non-fasting blood glucose levels and (**B**) body weight of male lean controls (open bars), and db/db (shaded bars) mice were measured at 3–20 weeks of age during T2D development. The black dotted lines define the normal blood glucose range for lean control males i.e., 3.85 mmol/l—11.65 mmol/l (mean ± 2 SD). Data show mean ± SEM for n = 5–13 mice/group. *p<0.05, **p<0.01 and ***p<0.001, compared to corresponding lean control group, Mann-Whitney test.
(TIF)

S3 Fig The intra-islet expression of HSPG core proteins rapidly declines in the islets of db/db female mice. Bar graphs show morphometric analysis of the % islets stained for the HSPG core proteins (**A**) COL18, (**B**) SDC1, (**C**) CD44 in pancreases of lean control female mice (wt, db/+; open bars) and db/db mice (shaded bars). Data show mean ± SEM for 3–6 pancreases/age group with n = 17–62 islets examined/group. *p<0.05, **p<0.01 and ***p<0.0001, Mann-Whitney test.
(TIF)

S4 Fig Distribution of HSPG core proteins and HS in the pancreatic islets of Ins2^WT/WT^ and Ins2^WT/C96Y^ islets. Representative images show intra-islet localisation of HSPG core proteins (**A, B**) COL18, (**C, D**) SDC1, (**E, F**) CD44 and (**G, H**) HS in Ins2^WT/WT^ (**A, C, E, G**) and Ins2^WT/C96Y^ (**B, D, F, H**) pancreas at 6 weeks of age. Scale bar = 100 μm.
(TIF)

S5 Fig Cell surface and intracellular expression of HSPG core proteins, HS and HPSE in MIN6 cells. Flow cytometry analysis of (**A**) HSPG core proteins COL18, SDC1 and CD44, (**B**) HS and (**C**) HPSE in MIN6 cells show significantly higher intracellular expression (shaded bars) of COL18, HS and HPSE compared to cell surface levels (open bars). Data represent geometric mean fluorescence intensity (GMFI) ± SEM, n = 4 independent experiments. *p<0.05, compared to corresponding cell surface expression, Mann-Whitney test.
(TIF)

S6 Fig Cell surface expression of HSPGs, HS and HPSE in wt and db/db isolated islet cells. (**A, D**) HSPG core proteins COL18, SDC1 and CD44, (**B, E**) HS and (**C, F**) HPSE in wt (open bars) and db/db (shaded bars) isolated islet cells show comparable weak cell surface expression at day 0 (upper panel) and after culture for 2 days (lower panel). The data show GMFI ± SEM, n = 3 experiments/group with n = 2–4 male donors/experiment. Mann-Whitney test.
(TIF)

S7 Fig HS replacement protects db/db beta cells from dying in culture. Representative flow cytometry dot plots show improved viability (Cal^+^PI^-^; upper left quadrant) of male (**A**) wild-type and (**B**) db/db beta cells (donors bg<10 mmol/l) after culture (**C, D**) without (Control) and (**E, F**) with heparin for 2 days. Cal^+^PI^+^ (upper right quadrant) identifies damaged cells; Cal^-^PI^+^ (lower right quadrant), dead cells; Cal^-^PI^-^ (lower left quadrant), cell debris. Each quadrant shows data as a % of the total cell population. n = 2–4 male islet donors/group.
(TIF)

S8 Fig Protection of wt beta cells *in vitro* using PI-88 as a HS replacer. Beta cells isolated from male (**A**) wt and (**B**) db/db (bg<15 mmol/l) donors were cultured without HS replacer (open bars) or with heparin (striped bars) or PI-88 (shaded bars) at 50 μg/ml for 2 days. (**A, B**) Cell viability (Cal^+^PI^-^) was examined on day 0 and day 2 by flow cytometry. Data show mean ± SEM; n = 3 experiments/group; n = 2–3 donor mice/experiment. **p<0.001, and ***p<0.0001, compared to corresponding controls, ANOVA with Fisher’s unprotected LSD post-test.
(TIF)

S9 Fig HS replacement renders male wt and db/db beta cells resistant to hydrogen peroxide (H_2_O_2_)-induced damage. Representative flow cytometry dot plots of Sytox Green uptake (boxed regions) show that H_2_O_2_ treatment of freshly isolated (**G**) wt and (**H**) db/db (donors bg<10 mmol/l) beta cells substantially increased beta cell damage/death compared to untreated controls on day 0 (**A, B**). Culture with heparin for 2 days protected (**C, D, I, J**) wt and (**E, F, K, L**) db/db beta cells from (**C-F**) culture-induced and (**I-L**) H_2_O_2_-induced damage/death. n = 2–4 male islet donors/experiment.
(TIF)

S1 File Percentage of islet area stained for HS, HSPGs (COL18, SDC1 and CD44) in male lean control (wt, db/+) and db/db pancreases. (XLSX)

S2 File Percentage of islet area stained for HSPGs (COL18, SDC1 and CD44) in female lean control (wt, db/+) and db/db pancreases. (XLSX)

S3 File Fold change in mRNA expression of ER stress associated genes in db/db islets. (XLSX)

S4 File (A, B) Fold change in the mRNA expression of ER stress associated genes in Akita Ins2^WT/C96Y^ islets; (C-F) The expression of HS and HSPG core proteins in the islets of Akita wild-type and Ins2^WT/C96Y^ pancreases. (XLSX)

S5 File (A) Fold change in the mRNA expression of ER stress associated genes in ER-stressed MIN6 beta cells; (B-F) Intracellular expression of HS, HSPGs and HPSE in control and ER- stressed MIN6 beta cells. (XLSX)

S6 File Geometric mean fluorescence intensity values for the expression of HSPG core proteins (COL18, SDC1 and CD44), HS and HPSE in wt and db/db islet cells. (XLSX)

S7 File (A, C, E, G) Viability of isolated wt and db/db mouse islet cells ± culture with HS mimetic (heparin); (B, D, F, H) Viability of isolated wt and db/db mouse islet cells ± culture with HS mimetic (heparin) and subsequent treatment with hydrogen peroxide. (XLSX)

S8 File (A, B) Blood glucose and body weight of saline- and TUDCA-treated db/db mice; (C) HbA1c levels of saline- and TUDCA-treated db/db mice; (D) Expression of ER stress associated genes in the islets of saline- and TUDCA-treated db/db mice; (E-G) Expression of HS, COL18 and insulin in the islets of saline- and TUDCA-treated db/db mice. (XLSX)
