# Supplementary material for: Heparan sulfate proteoglycans in beta cells provide a critical link between endoplasmic reticulum stress, oxidative stress and type 2 diabetes
Source: PLoS One. 2021 Jun 4;16(6):e0252607. doi: 10.1371/journal.pone.0252607 (PMC8177513; doi:10.1371/journal.pone.0252607)
Supplement: S1 Table — (DOCX) [file pone.0252607.s001.docx]

**S1 Table.** **Primer/probe set for TaqMan RT-PCR.**

| **Gene name** | **Gene symbol** | **Taqman gene expression assay ID^a^** |
| --- | --- | --- |
| Ubiquitin conjugating enzyme E2D 1 | Ube2d1 | Mm00461037_g1 |
| Glyceraldehyde-3-phosphate dehydrogenase | Gapdh | Mm99999915_g1 |
| Heat shock protein 5 | Hspa5, Bip | Mm00517690_g1 |
| DnaJ (Hsp40) homolog, subfamily C, member 3 | Dnajc3, p58 | Mm00515299_m1 |
| DNA-damage inducible transcript 3 | Ddit3, Chop | Mm01135937_g1 |
| Activating transcription factor 3 | Atf3 | Mm00476032_m1 |

^a^ ThermoFisher Scientific.
