# Supplementary material for: Heparan sulfate proteoglycans in beta cells provide a critical link between endoplasmic reticulum stress, oxidative stress and type 2 diabetes
Source: PLoS One. 2021 Jun 4;16(6):e0252607. doi: 10.1371/journal.pone.0252607 (PMC8177513; doi:10.1371/journal.pone.0252607)
Supplement: S2 Table — (DOCX) [file pone.0252607.s002.docx]

**S2 Table.** **Antibodies used for flow cytometry.**

| **Name** | **Code** | **Stock conc.** | **Source** | **Final conc.** |
| --- | --- | --- | --- | --- |
| Rat anti-mouse CD16/CD32 (mouse Fc block) | 553142 | 0.5 mg/ml | BD Biosciences | 5 μg/ml |
| Rat anti- mouse CD138 (SDC1) | 553712 | 0.5 mg/ml | BD Biosciences | 20 μg/ml |
| Rat anti-mouse CD44 (CD44) | 553130 | 1 mg/ml | BD Biosciences | 40 μg/ml |
| Mouse anti-human heparan sulfate F58-10E4 | 370255-1 | 1 mg/ml | Seikagaku Corporation; Amsbio | 20 μg/ml |
| Mouse anti-mouse COL18A1 (COL18) | 1837-46 | 200 µg/ml | Santa Cruz Biotechnology | 4 μg/ml |
| Mouse anti-human heparanase HP3/17 | INS-26-1-0000-12 | 150 μg/ml | Insight Biopharmaceuticals | 1.5 μg/ml |
| Goat anti-mouse Ig PE | 1012-09 | 0.25 mg/ml | Southern Biotech | 2.5 or 5 μg/ml |
| Mouse anti-rat kappa PE | 3090-09 | 0.1 mg/ml | Southern Biotech | 2 μg/ml |
| Rat anti-mouse Ig FITC | 553395 | 0.5 mg/ml | BD Biosciences | 10 μg/ml |
