# Supplementary material for: Heparan sulfate proteoglycans in beta cells provide a critical link between endoplasmic reticulum stress, oxidative stress and type 2 diabetes
Source: PLoS One. 2021 Jun 4;16(6):e0252607. doi: 10.1371/journal.pone.0252607 (PMC8177513; doi:10.1371/journal.pone.0252607)
Supplement: S3 Table — (DOCX) [file pone.0252607.s003.docx]

**S3 Table.** **Antibodies used for immunohistochemistry.**

| **Name** | **Code** | **Stock conc.** | **Source** | **Final conc.** |
| --- | --- | --- | --- | --- |
| Rat anti-mouse CD138 (SDC1) | 553712 | 0.5 mg/ml | BD Biosciences | 50 µg/ml |
| Rat anti-mouse CD44 (IM7) | 553130 | 1 mg/ml | BD Biosciences | 40 µg/ml |
| Mouse anti-human CD44v3 (CD44v3) | BBA11 | 0.5 mg/ml | R&D systems | 10 µg/ml |
| Mouse anti-mouse COL18A1 (COL18) | 1837-46 | 200 µg/ml | Santa Cruz Biotechnology | 4 µg/ml |
| Mouse anti-human HS, F58-10E4 (HS) | 370255-1 | 1 mg/ml | Amsbio | 200 µg/ml |
| Phage display single chain EV3C3 anti-HS | ^a^Dr Toin Van Kuppervelt | | | |
| Mouse anti-insulin (ascites) | I2018 | 32.5216 mg/ml | Sigma-Aldrich | 65 or 260 µg/ml |
| Purified rat IgG_2aκ_ | 559073 | 0.5 mg/ml | BD Biosciences | 50 µg/ml |
| Purified NA/LE rat IgG_2bκ_ | 555845 | 1 mg/ml | BD Biosciences | 40 µg/ml |
| Purified mouse IgG_2b_ | 557351 | 0.5 mg/ml | BD Biosciences | 4 or 10 µg/ml |
| Purified mouse IgM_κ_ | 550340 | 250 µg/ml | BD Biosciences | 200 µg/ml |
| Mouse IgG_1κ_ | 14-4714-85 | 0.5 mg/ml | eBioscience | 64.8 or 227.3 µg/ml |
| Polyclonal rabbit anti-rat immunoglobulins/ HRP (for SDC1 and CD44 (IM7)) | P0450 | 1.3 g/l | DAKO | 52 µg/ml |
| Polyclonal rabbit anti-mouse immunoglobulins/HRP (for COL18 and HS) | P0161 | 1.3 g/l | DAKO | 26 µg/ml |
| Polyclonal rabbit anti-VSV-G | V4888 | 1 mg/ml | Sigma-Aldrich | 10 µg/ml |
| Polyclonal swine anti-rabbit immunoglobulins/HRP (for EV3C3 anti-HS) | P0217 | 1.3 g/l | DAKO | 13 µg/ml |
| Vector M.O.M biotinylated anti-mouse IgG (for insulin) | PK-2200 | Unknown | Vector Laboratories Inc. | 1/250 dilution |
| HRP-rabbit anti-mouse IgG (for CD44v3) | 31450 | 0.8 mg/ml | Invitrogen | 8 µg/ml |
| HRP-rabbit anti-rat IgG (alternative for CD44 (IM7)) | A5795 | 7.4 mg/ml | Invitrogen | 0.74 mg/ml |

^a^A gift from Dr Toin Van Kuppervelt, University of Nijmegen, The Netherlands
