# Supplementary material for: Heparan sulfate proteoglycans in beta cells provide a critical link between endoplasmic reticulum stress, oxidative stress and type 2 diabetes
Source: PLoS One. 2021 Jun 4;16(6):e0252607. doi: 10.1371/journal.pone.0252607 (PMC8177513; doi:10.1371/journal.pone.0252607)
Supplement: S4 Table — (DOCX) [file pone.0252607.s004.docx]

**S4 Table.** **Cell surface and intracellular expression of HSPG core proteins, HS and HPSE in wt and db/db mouse beta cells.**

| **HSPGs/HS/HPSE** | **Culture^a^** | **wt beta cells^b^** | | **db/db beta cells^b^** | |
| --- | --- | --- | --- | --- | --- |
|  |  | **Intracellular** | **Cell surface** | **Intracellular** | **Cell surface** |
| **COL18** | **Day 0**  **Day 2** | 3848.0±575.1  5432.0±732.7 | 112.4±20.4*  140.8±32.2* | 1780.4±148.7  941.9±142.8^##^ | 143.3±22.9*  149.1±30.8* |
| **SDC1** | **Day 0**  **Day 2** | 354.4±89.9  632.0±212.7 | 93.7±27.9*  192.6±148.1 | 131.1±15.2  174.8±42.2 | 115.9±72.3  353.7±297.7 |
| **CD44** | **Day 0**  **Day 2** | 326.2±34.5  299.2±18.1 | 103.1±7.37*  170.3±17.6* | 352.5±29.9  248.8±24.6 | 150.3±7.4*  260.7±42.0 |
| **HS** | **Day 0**  **Day 2** | 1000.4±129.2  1279.3±213.9 | 124.5±26.8*  160.3±45.2* | 818.1±95.8  479.6±60.6^##^ | 175.7±26.4*  188.5±46.3* |
| **HPSE** | **Day 0**  **Day 2** | 1649.6±180.2  1792.1±233.2 | 130.6±10.4*  396.3±75.3* | 775.3±66.2  474.3±95.7^#^ | 131.0±6.1*  409.3±126.6 |

^a^ Mouse beta cells were analysed on day 0 and day 2 by flow cytometry (see Materials and Methods).

^b^ Data for GMFI show Mean±SEM

**p*<0.05 comparison between intracellular and corresponding cell surface expression; Mann-Whitney test

^#^*p*<0.05, ^##^*p*<0.01 comparison between day 0 and day 2 for db/db beta cells; Mann-Whitney and Unpaired t test
