# Supplementary figures and images for: Heparan sulfate proteoglycans in beta cells provide a critical link between endoplasmic reticulum stress, oxidative stress and type 2 diabetes
Source: PLoS One. 2021 Jun 4;16(6):e0252607. doi: 10.1371/journal.pone.0252607 (PMC8177513; doi:10.1371/journal.pone.0252607)

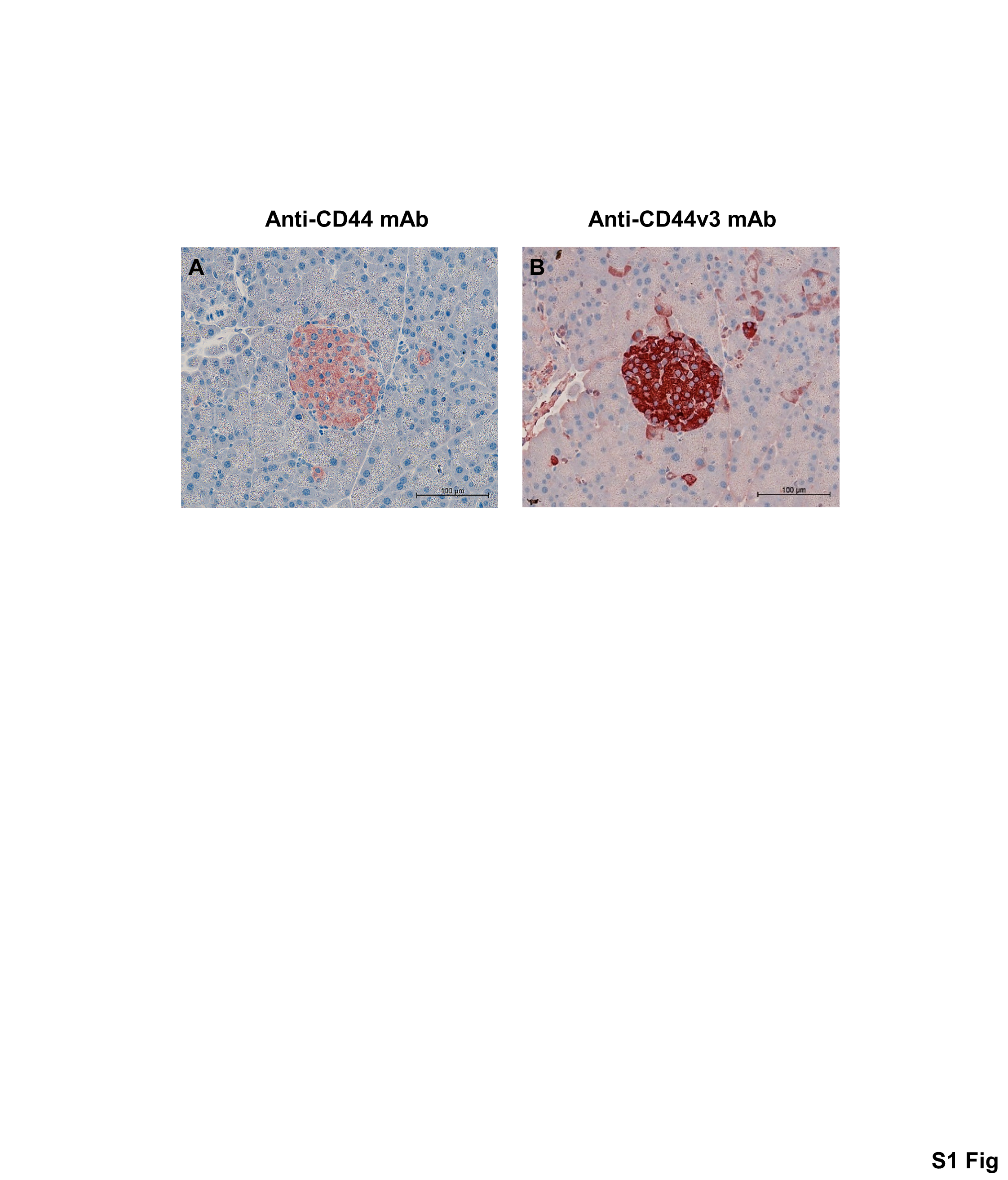

Supplement: S1 Fig — Immunostaining of the same islet in wt (+/+) pancreas (9 weeks of age) shows similar localization of CD44 using (A) rat anti-mouse CD44 (IM7) mAb and (B) mouse anti-human CD44v3 mAb. Scale bar = 100 μm. (TIF) [file pone.0252607.s005.tif]

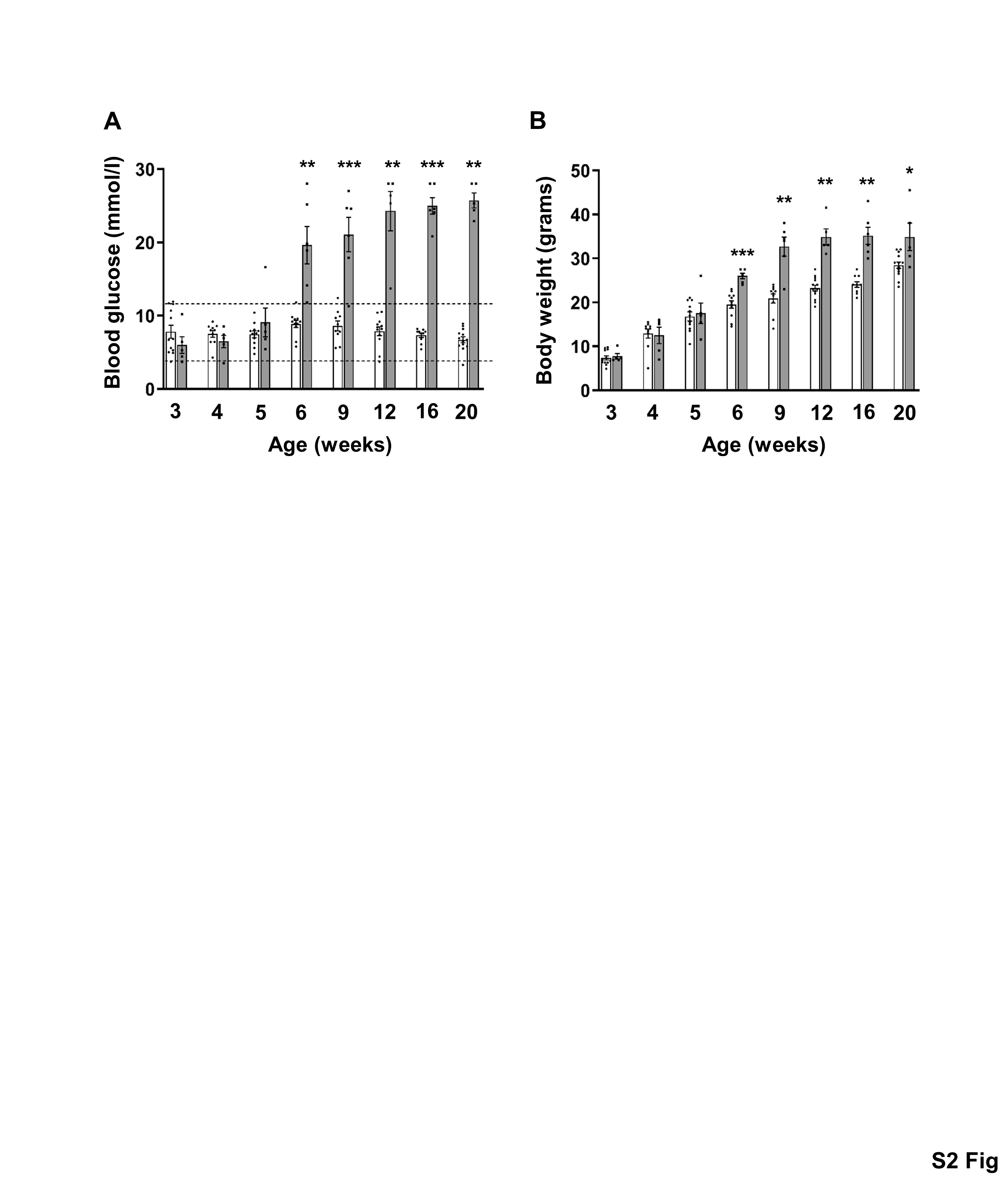

Supplement: S2 Fig — (A) Non-fasting blood glucose levels and (B) body weight of male lean controls (open bars), and db/db (shaded bars) mice were measured at 3–20 weeks of age during T2D development. The black dotted lines define the normal blood glucose range for lean control males i.e., 3.85 mmol/l—11.65 mmol/l (mean ± 2 SD). Data show mean ± SEM for n = 5–13 mice/group. *p<0.05, **p<0.01 and ***p<0.001, compared to corresponding lean control group, Mann-Whitney test. (TIF) [file pone.0252607.s006.tif]

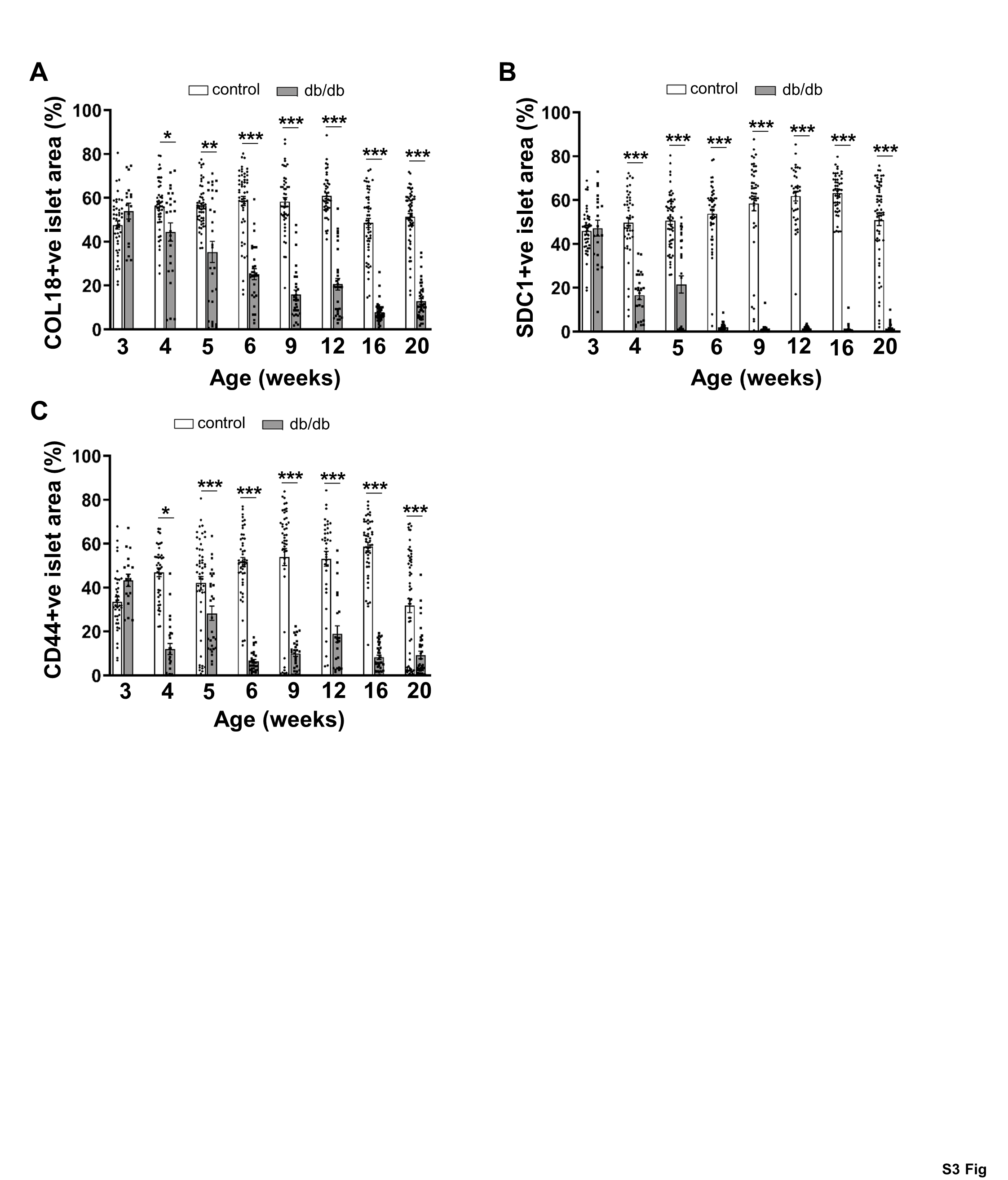

Supplement: S3 Fig — Bar graphs show morphometric analysis of the % islets stained for the HSPG core proteins (A) COL18, (B) SDC1, (C) CD44 in pancreases of lean control female mice (wt, db/+; open bars) and db/db mice (shaded bars). Data show mean ± SEM for 3–6 pancreases/age group with n = 17–62 islets examined/group. *p<0.05, **p<0.01 and ***p<0.0001, Mann-Whitney test. (TIF) [file pone.0252607.s007.tif]

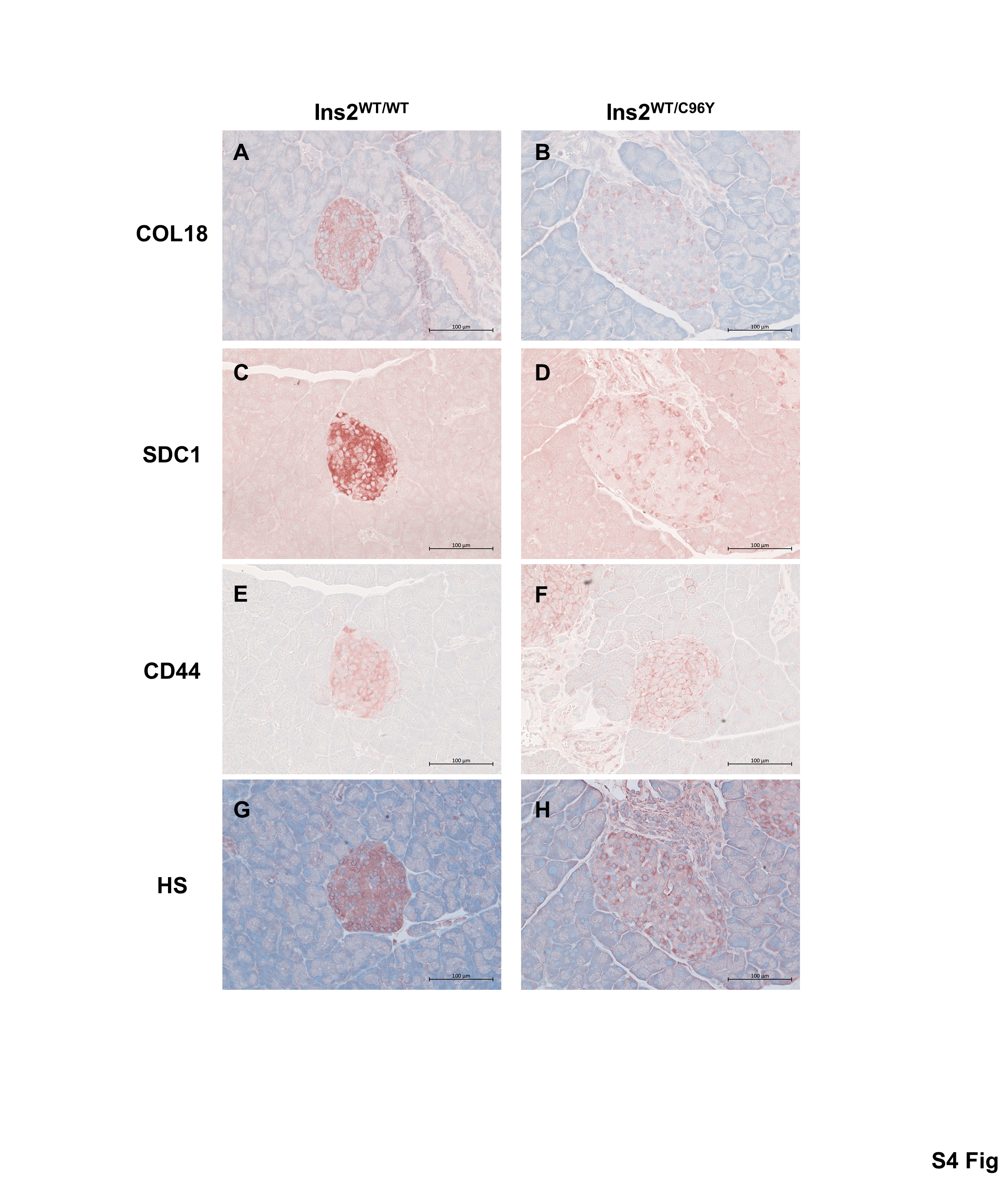

Supplement: S4 Fig — Representative images show intra-islet localisation of HSPG core proteins (A, B) COL18, (C, D) SDC1, (E, F) CD44 and (G, H) HS in Ins2WT/WT (A, C, E, G) and Ins2WT/C96Y (B, D, F, H) pancreas at 6 weeks of age. Scale bar = 100 μm. (TIF) [file pone.0252607.s008.tif]

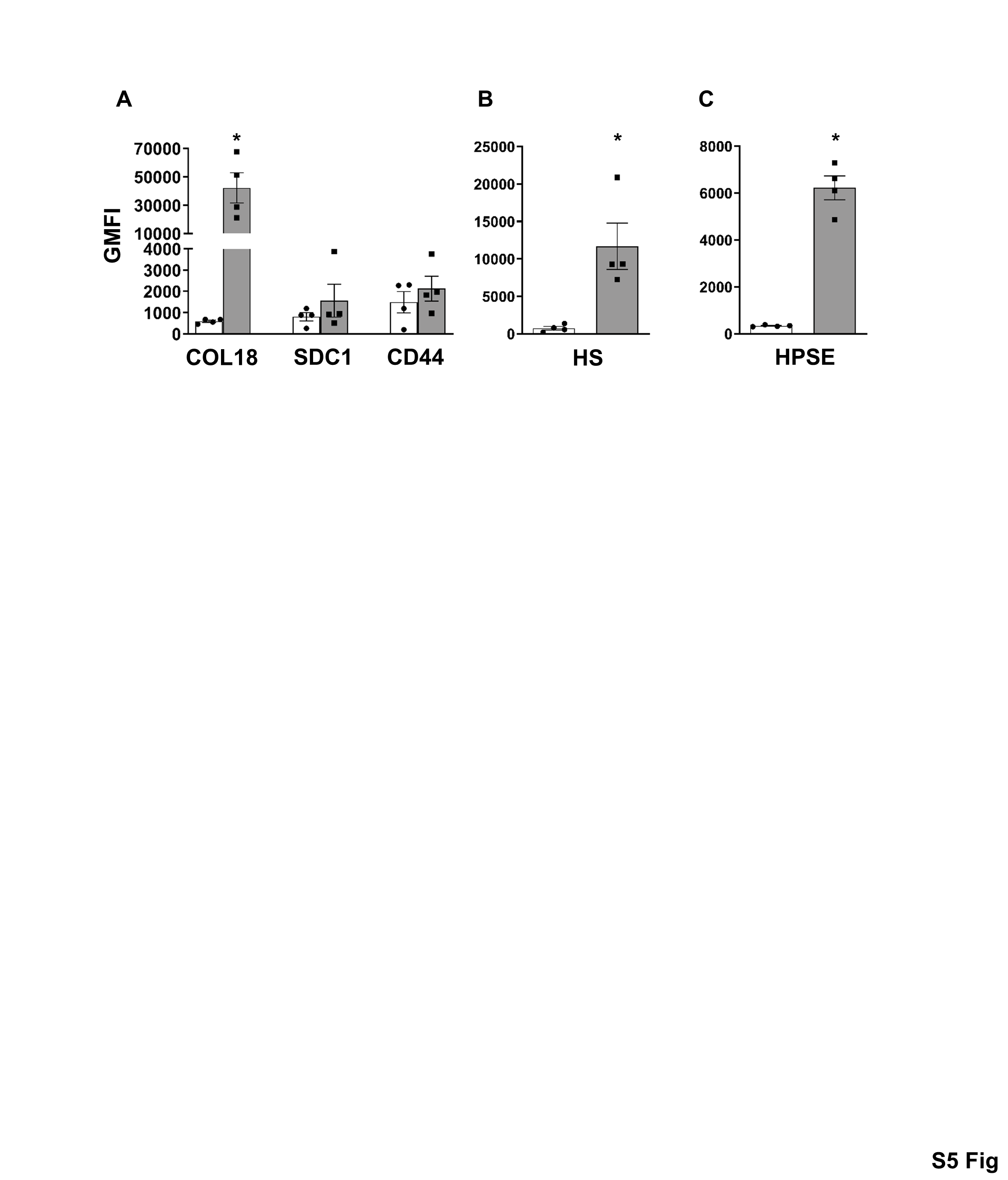

Supplement: S5 Fig — Flow cytometry analysis of (A) HSPG core proteins COL18, SDC1 and CD44, (B) HS and (C) HPSE in MIN6 cells show significantly higher intracellular expression (shaded bars) of COL18, HS and HPSE compared to cell surface levels (open bars). Data represent geometric mean fluorescence intensity (GMFI) ± SEM, n = 4 independent experiments. *p<0.05, compared to corresponding cell surface expression, Mann-Whitney test. (TIF) [file pone.0252607.s009.tif]

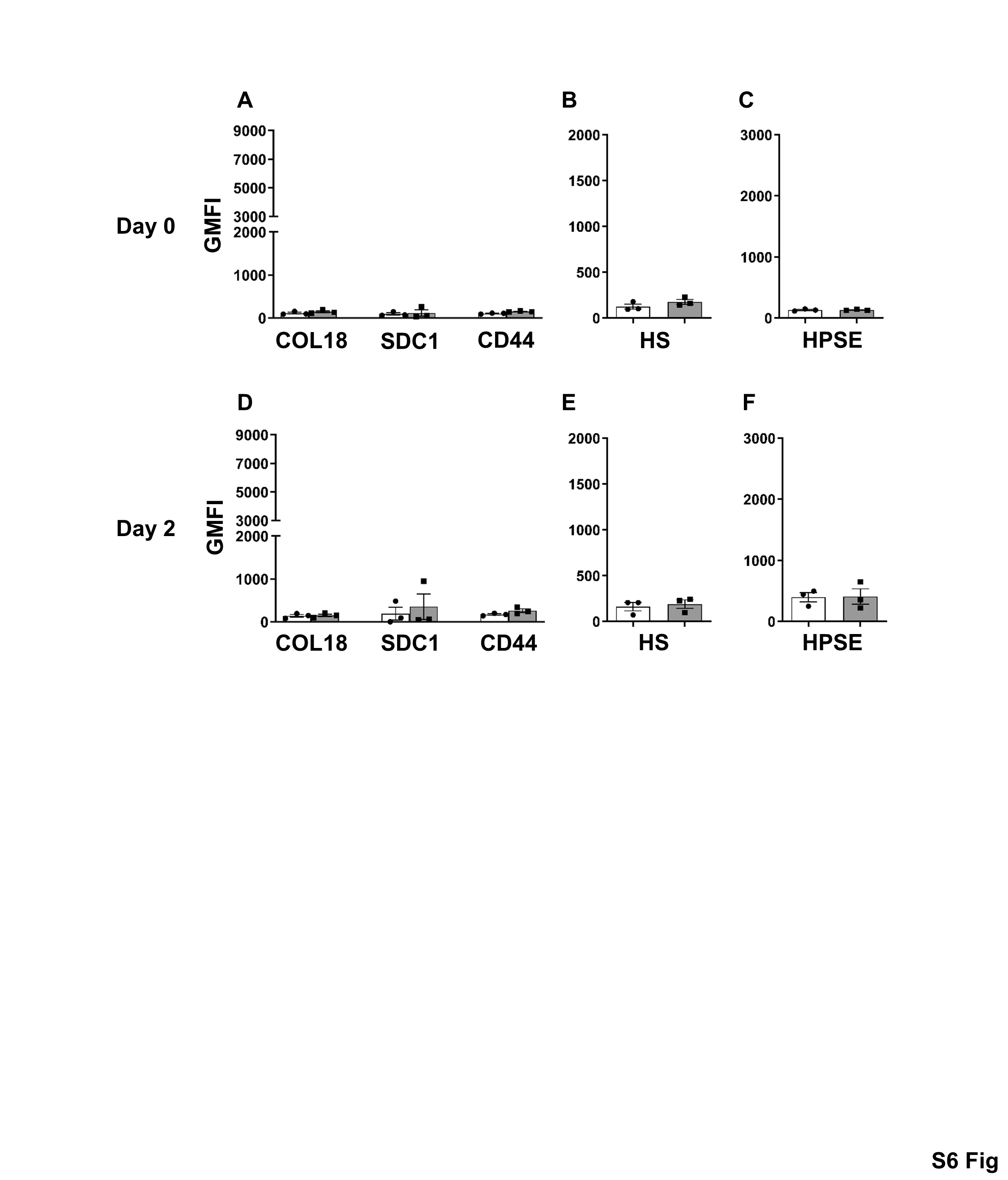

Supplement: S6 Fig — (A, D) HSPG core proteins COL18, SDC1 and CD44, (B, E) HS and (C, F) HPSE in wt (open bars) and db/db (shaded bars) isolated islet cells show comparable weak cell surface expression at day 0 (upper panel) and after culture for 2 days (lower panel). The data show GMFI ± SEM, n = 3 experiments/group with n = 2–4 male donors/experiment. Mann-Whitney test. (TIF) [file pone.0252607.s010.tif]

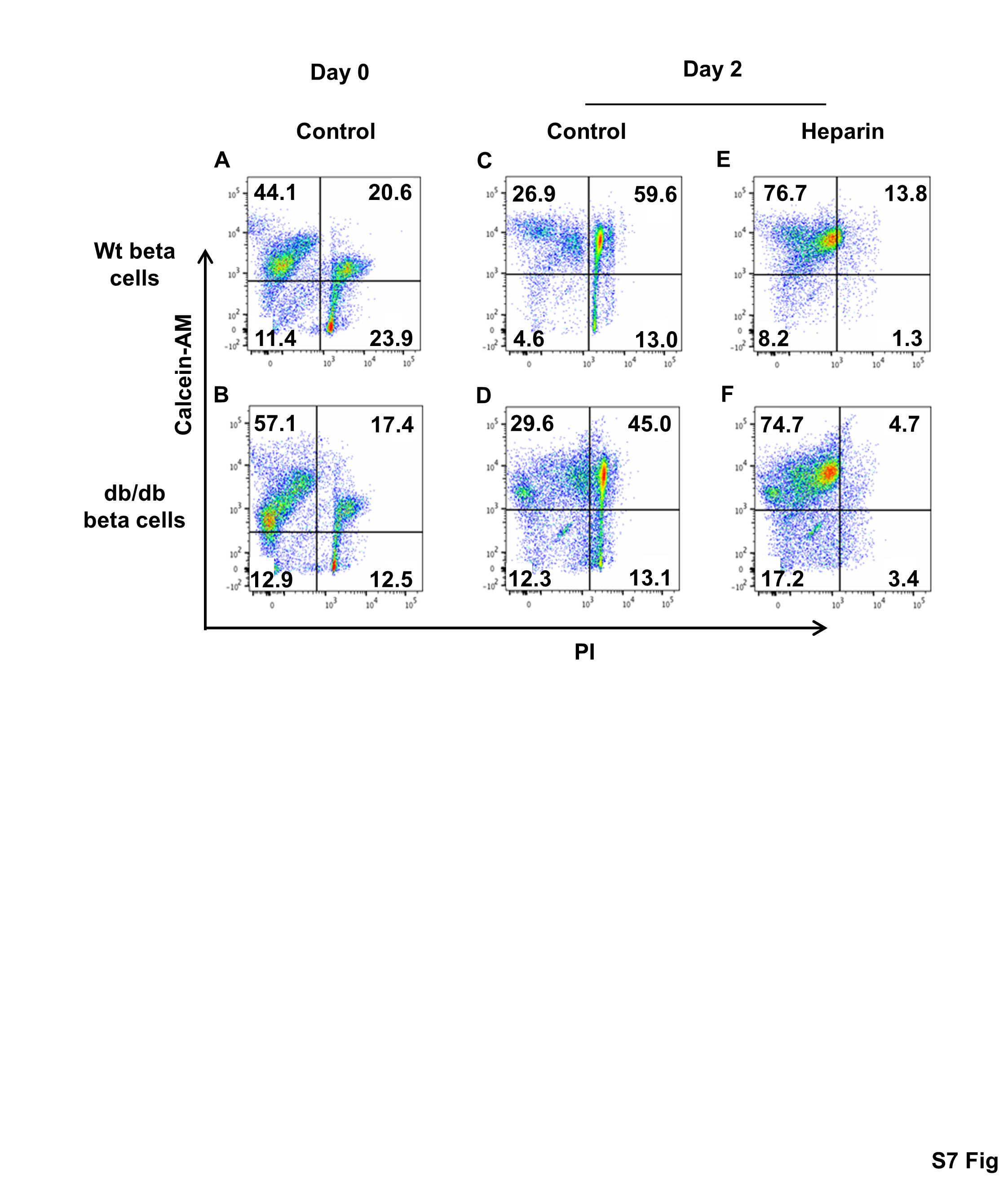

Supplement: S7 Fig — Representative flow cytometry dot plots show improved viability (Cal+PI-; upper left quadrant) of male (A) wild-type and (B) db/db beta cells (donors bg<10 mmol/l) after culture (C, D) without (Control) and (E, F) with heparin for 2 days. Cal+PI+ (upper right quadrant) identifies damaged cells; Cal-PI+ (lower right quadrant), dead cells; Cal-PI- (lower left quadrant), cell debris. Each quadrant shows data as a % of the total cell population. n = 2–4 male islet donors/group. (TIF) [file pone.0252607.s011.tif]

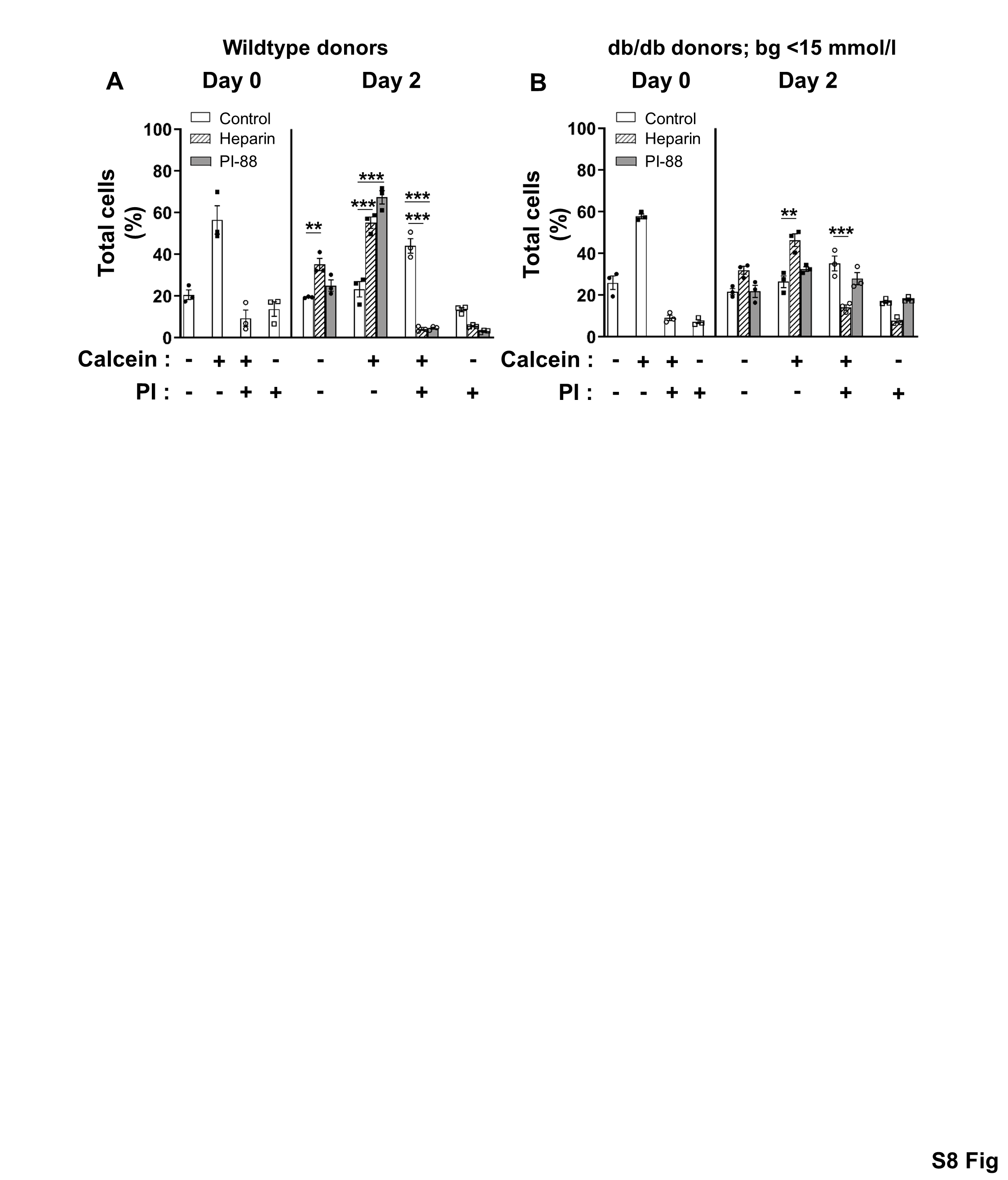

Supplement: S8 Fig — Beta cells isolated from male (A) wt and (B) db/db (bg<15 mmol/l) donors were cultured without HS replacer (open bars) or with heparin (striped bars) or PI-88 (shaded bars) at 50 μg/ml for 2 days. (A, B) Cell viability (Cal+PI-) was examined on day 0 and day 2 by flow cytometry. Data show mean ± SEM; n = 3 experiments/group; n = 2–3 donor mice/experiment. **p<0.001, and ***p<0.0001, compared to corresponding controls, ANOVA with Fisher’s unprotected LSD post-test. (TIF) [file pone.0252607.s012.tif]

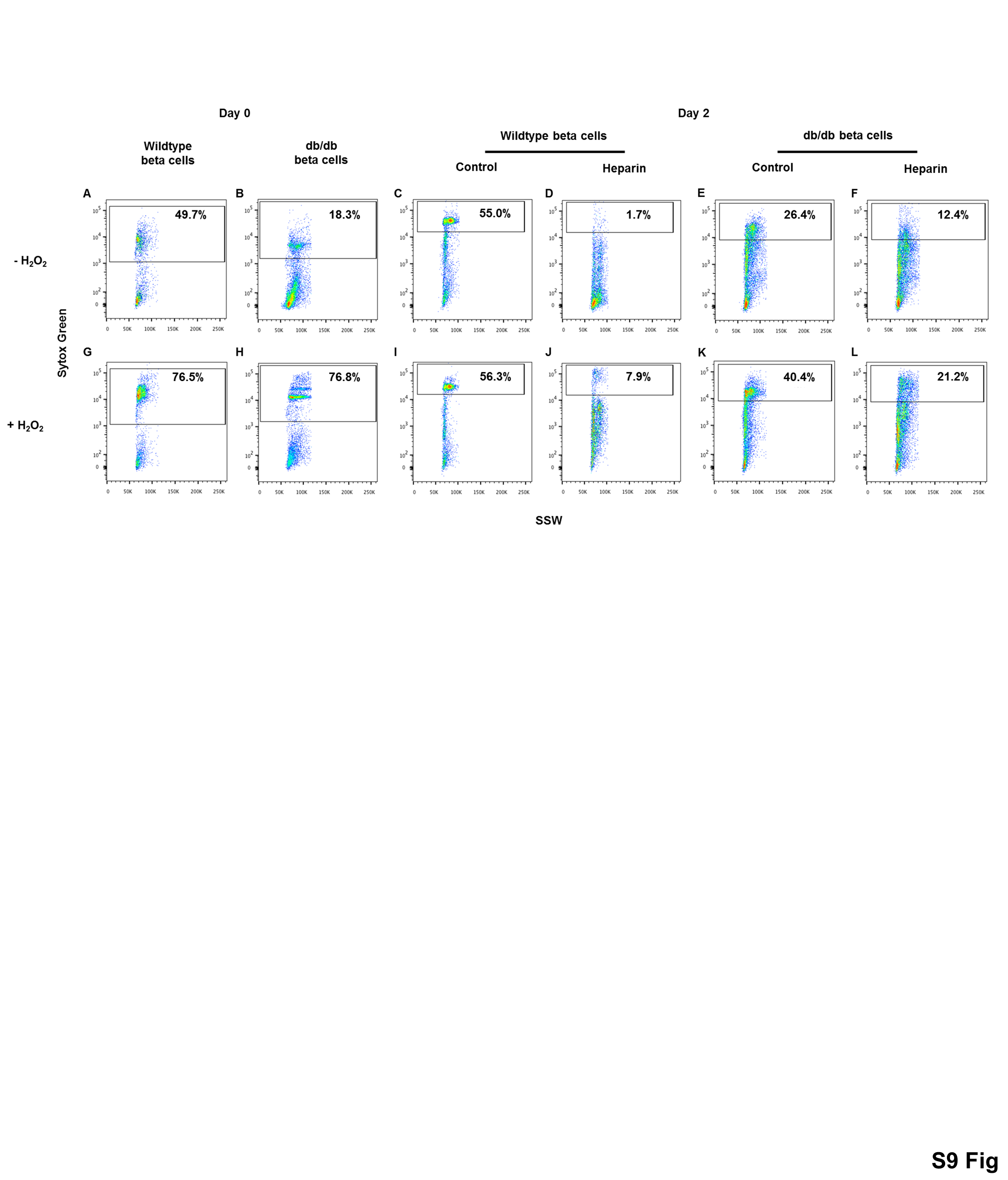

Supplement: S9 Fig — Representative flow cytometry dot plots of Sytox Green uptake (boxed regions) show that H2O2 treatment of freshly isolated (G) wt and (H) db/db (donors bg<10 mmol/l) beta cells substantially increased beta cell damage/death compared to untreated controls on day 0 (A, B). Culture with heparin for 2 days protected (C, D, I, J) wt and (E, F, K, L) db/db beta cells from (C-F) culture-induced and (I-L) H2O2-induced damage/death. n = 2–4 male islet donors/experiment. (TIF) [file pone.0252607.s013.tif]
